# Microbial community diversity from nearshore to offshore in the East China Sea

**DOI:** 10.3389/fmicb.2024.1377001

**Published:** 2024-05-27

**Authors:** Jian Jin, Xiujie Liu, Wenbin Zhao, Hao Sun, Siyin Tan, Xiao-Hua Zhang, Yunhui Zhang

**Affiliations:** ^1^Frontiers Science Center for Deep Ocean Multispheres and Earth System, College of Marine Life Sciences, Ocean University of China, Qingdao, China; ^2^Key Laboratory of Evolution and Marine Biodiversity (Ministry of Education), Institute of Evolution and Marine Biodiversity, Ocean University of China, Qingdao, China; ^3^Laboratory for Marine Ecology and Environmental Science, Laoshan Laboratory, Qingdao, China

**Keywords:** microbial community, co-occurrence pattern, seasonality, community assembly, the PN section, East China Sea

## Abstract

The Pollution Nagasaki (PN) section of the East China Sea (ECS) is a typical area for studying the complex hydrographic dynamics between Changjiang River discharge and Kuroshio, displaying intense variations of environmental gradients from nearshore to offshore. However, the temporal and spatial changes of microbial communities along the PN section have long been overlooked. In this study, we performed a comprehensive investigation into the abundance, diversity and ecology of free-living (FL) and particle-associated (PA) microbial communities in seawater samples along the PN section during both summer and winter. Distinct hydrological conditions and resulting environmental gradients were observed between summer and winter, with clear features of intrusive Kuroshio subsurface water in summer and strong vertical mixing of seawater in winter. Bacterial abundance along the PN section was higher in summer (1.11 × 10^8^ copies·L^−1^ – 7.37 × 10^8^ copies·L^−1^) than in winter (1.83 × 10^6^ copies·L^−1^ – 1.34 × 10^8^ copies·L^−1^). Microbial diversity, as indicated by α-diversity indices, remained at relatively stable levels in summer, while a clear decreasing trend was observed in winter along the PN section. Additionally, the winter communities exhibited a more evident spatial shift along the PN section compared to the summer communities. 16S rRNA gene amplicon sequencing showed that microbial community composition varied considerably between different seasons (summer and winter) and lifestyles (FL and PA), with a notable dominance of *Ralstonia* species. in winter. Regarding the assembly of microbial communities, the stochastic process represented by dispersal limitation was the dominant process in summer, while the deterministic homogeneous selection was the most important process in winter. Correspondingly, distinct topological properties of the microbial co-occurrence networks were shown between different seasons and along the PN section. These results enhance our understanding of how hydrological conditions influence dynamic changes of microbial communities along the PN section, providing new insights for the microbial community assembly and interactions in such a complex environment.

## Introduction

The East China Sea (ECS) is the largest marginal sea of the northwest Pacific Ocean, bordered by the coast of China to the west and the Korean Peninsula and Japan to the east (Wong et al., 2000; Yue et al., 2021). The coastal area of the ECS is subject to intricate hydrodynamic processes, including the Changjiang (Yangtze) River discharge and Kuroshio water intrusion (Zhou et al., 2019). Substantial terrestrial substances and freshwater can be carried by the Changjiang River into the ESC, while the warm and saline Kuroshio water exchanges actively with the ECS shelf water (Li et al., 2016; Liu et al., 2021). The Pollution Nagasaki (PN) section traverses the middle of the ECS shelf from 30°30′N, 124°30′E in the northwest to 27°30′N, 128°15′E in the east, which starts off the Changjiang Estuary and ends at Ryukyu Islands with significant changes in water depth (from 100 m to 1,000 m) (Siswanto et al., 2007; Qian et al., 2023). This section not only passes through the freshwater area of the Changjiang River, but also intersects the Okinawa Trough and is perpendicular to the main axis of the Kuroshio, making it a representative area for investigating the hydrographic, chemical and ecological characteristics of the ECS (Liu, 2001). The Kuroshio is the Pacific western boundary current bypassing the Luzon Strait and entering the ECS through the East Taiwan Channel. It serves as an important carrier of heat and salt into the ESC (Hsueh, 2000). With different characteristics in temperature, salinity and nutrient inputs, the interactions between Changjiang river discharge and Kuroshio intrusion lead to the formation of complex water masses. Correspondingly, microorganisms along the PN section can be highly variable due to the environmental heterogeneity such as the nutrient sources and hydrodynamic conditions that significantly influence the distribution of microbial communities and related biogeochemical processes. Despite many studies focusing on the dynamics of hydrological changes, nutrient inputs and the phytoplankton communities in the PN section (Furuya et al., 2003; Pan et al., 2005; Zhu et al., 2005; Guo et al., 2013; Liu et al., 2021; Zhang et al., 2021, 2022), the temporal and spatial changes of microbial communities (including their abundance, diversity and ecology) in this complex area have long been overlooked.

Elucidating microbial community dynamics and drivers in the coastal ocean is critical for the marine biogeochemical cycle, given the high productivity in coastal margins (Lucas et al., 2011). Changes in these microbial communities are important and direct indicators of sharp environmental gradient transitions from the nearshore to the open ocean (Fortunato et al., 2012). Understanding coastal microbial communities is further complicated by temporal environmental changes, including physical oceanographic features, the seasonality of environmental parameters, as well as interactions with other organisms (Zhu et al., 2023). Key environmental factors associated with marine microbial community composition including salinity, temperature, light, depth, primary productivity and distinct water masses etc. (Gilbert et al., 2012; Giovannoni and Vergin, 2012; Yung et al., 2016). While these environmental factors are considered deterministic processes in ecological theory, the community structure of microorganisms is also influenced by stochastic processes such as dispersal and ecological drift (Stegen et al., 2012). The extent to which these ecological processes shape microbial distribution patterns and their relative importance in determining microbial community assembly along the PN section in the coastal ECS remains largely unknown. Moreover, microbial interactions, as inferred by the correlation-based networks, play a crucial role in maintaining a diverse microbial (Faust and Raes, 2012). The sharp environmental gradients and complex hydrodynamic conditions may lead to distinct co-occurrence patterns of seawater microbial communities in the PN section.

Considering the significance of the PN section as a typical area for studying the physical, chemical and biological oceanographic interactions between Changjiang plume front, shelf water and Kuroshio (Wu et al., 2003), as well as the lack of in-depth research on the spatial and temporal changes of microbial communities along the PN section, we collected water samples from different depths in summer (June 2018) and winter (December 2019). The free-living (FL) and particle-associated (PA) microbial counterparts which may differ significantly in diversity, community and their response to the environment (D'Ambrosio et al., 2014) in these samples were investigated. Based on detailed profiling of crucial environmental factors, we inferred the possible hydrodynamic processes in different seasons, and further studied the microbial abundance, diversity, compositions, assembly processes and interactions in the PN section. The main objectives of the current study were to reveal (1) the spatial and temporal changes of microbial communities and diversity in the PN section, along with the driving environmental factors; (2) the differences in microbial assembly processes and associations in distinct seasons along the PN section. Our findings provide novel insights for understanding the complex hydrological changes and microbial community dynamics in this ecologically important region.

## Materials and methods

### Sampling and environmental parameters

**Figure 1:**
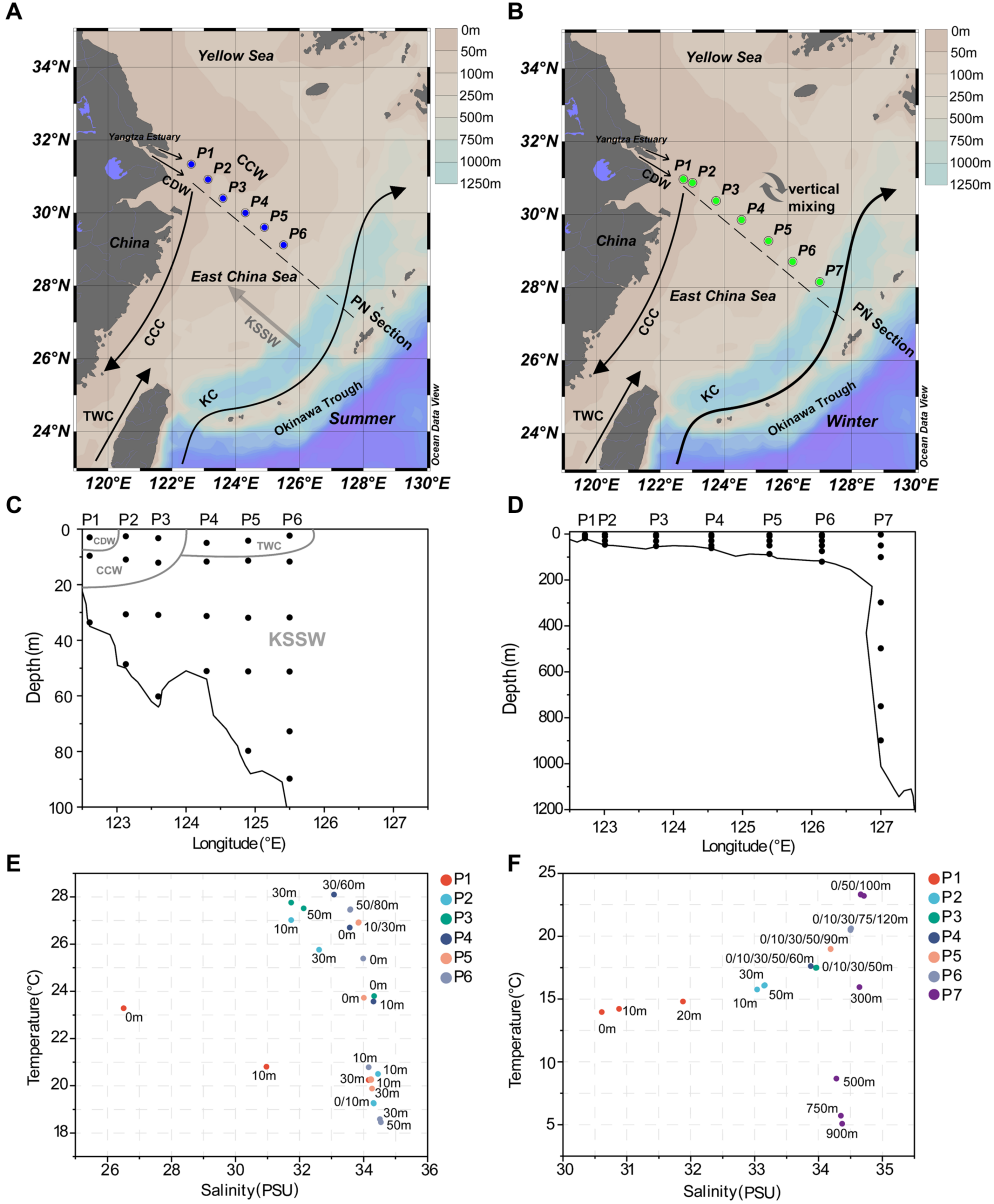
Sampling information along the PN section. **(A)** Sampling sites in summer; **(B)** sampling sites in winter; **(C)** the sampling depth in summer; **(D)** the sampling depth in winter; **(E)** scatterplot of salinity-temperature for summer samples; **(F)** scatterplot of salinity-temperature for winter samples. CDW, Changjiang Diluted Water; TWC, Taiwan Warm Current; CCC, China Coast Current; KC, Kuroshio Current; CCW, Continental Coastal Water; KSSW, Kuroshio Subsurface Water.

The seawater samples were collected from 6 sites aboard R/V *Dongfang Hong* 2 in June 2018 (Figures 1A,C) and 7 sites aboard R/V *Dongfang Hong* 3 in December 2019 (Figures 1B,D), respectively. Summer seawater samples from the PN section were collected from 122.61–125.50°E, 29.12–31.33°N, while winter seawater samples were collected from 122.72–127.60°E, 28.15–30.96°N. The seawater samples were collected using a Sealogger CTD (SBE25, Electronic Inc., United States) rosette water sampler. Samples containing 1 L seawater were filtered serially through 3 μm and 0.22 μm polycarbonate membranes (Millipore Corporation, Billerica, MA, United States), respectively. The collections above 3 μm and between 0.22 μm and 3 μm were considered as the PA and FL samples, respectively. The detailed information of 52 summer samples (26 FL and 26 PA) and 68 winter samples (34 FL and 34 PA) in total are shown in Supplementary Tables S1, S2. In addition, 2 mL of water were taken from each sample into a sterile tube and were immediately fixed with paraformaldehyde (final concentration 4%, v/v) for 30 min in the dark at room temperature, and were used for subsequent determination of absolute abundance of eukaryotes and prokaryotes. Membranes and 2 mL water samples were frozen in liquid nitrogen immediately and stored at −20°C on board before transferred into −80°C in laboratory.

For environmental parameters, temperature, salinity and depth were obtained by CTD equipped on the water sampler *in situ*. The concentrations of Chlorophyll *a* (Chl *a*) were measured as described by Zhang Y. et al. (2014). Briefly, seawater samples were filtered by using GF/F filter with a pore size of 0.7 μm immediately after collection on board, and then soaked in 90% (v/v) acetone in the dark for 24 h to extract Chl *a*. The concentrations of Chl *a* in the extract were determined using a F4500 fluorescence spectrophotometer (Hitachi, Japan). The dissolved oxygen (DO) was measured by the Winkler method (Carpenter, 1965). Samples for dissolved inorganic nutrients (PO_4_^3−^, NO_2_^−^, NO_3_^−^, SiO_3_^2−^ and NH_4_^+^) were filtered with 0.45 μm cellulose acetate membranes and were analyzed by an Auto-Analyzer (AA3, Seal Analytical Ltd., UK) (Liu et al., 2015). The abundances of *Synechococcus* (SYN), *Prochlorococcus* (PRO), picoeukaryotes (PEUK), and heterotrophic bacteria (HB) were measured by flow cytometer (BD FACSJazz, United States). SYN, PRO and PEUK were detected directly by their autofluorescence and size (SYN: orange and < 2 μm, PRO: red and < 2 μm, PEUK: red and > 2 μm). For HB, 50 μL seawater samples are diluted with 250 μL TE buffer solution (Tris EDTA, 100 mM Tris-Cl, 10 mM EDTA, pH = 8.0, Sigma, United States), and 4 μL nucleic acid dye SYBR Green I (Molecular Probes, United States) was added to stain for 20 min before detection (Zhao et al., 2012, 2017).

### Total DNA extraction and quantitative PCR

Total DNA were extracted from 52 summer and 68 winter samples using the Phenol-chloroform method (Yin et al., 2013; Sun et al., 2020). The extracted DNA was dissolved in 10 mM Tris–HCl (pH 8.0) and stored at −80°C for subsequent 16S rRNA gene amplicon sequencing and qPCR.

The abundances of total bacterial 16S rRNA genes in seawater samples were quantified by qPCR with the primer set 338F (5′-ACTCCTACGGGAGGCAGCAG-3′) and 518R (5′-ATTACCGC GGCTGCTGG-3′) (Yin et al., 2013) on StepOne^™^ Real-time PCR System (Applied Biosystems). The PCR reactions, standard curves and melting curves were conducted as previous described by Sun et al. (2020). According to the standard curves, the detection range of qPCR was 5.02 × 10^5^ copies L^−1^- 5.02 × 10^11^ copies L^−1^. The specific PCR reactions were as follows: an initial denaturation at 95°C for 3 min, then 35 cycles of 95°C for 30s, primer- specific annealing temperature 53°C for 30 s, 72°C for 30 s. The qPCR gene amplification efficiency ranged from 95 to 105%. Three techniques replicates were set for each sample. Double-distilled water was used as the template for negative control.

### Sequencing and microbial community analysis

The 16S rRNA gene amplicon sequencing was conducted by Majorbio Bio-Pharm Technology Co. Ltd. (Shanghai, China). The hypervariable V4 region of the bacterial and archaeal 16S rRNA gene was amplified by using primers 515FmodF (5′-GTGYCAGCMG CCGCGGTAA-3′) and 806RmodR (5′-GGACTACNVGGGTW TCTAAT-3′) (Walters et al., 2016). The PCR amplification was conducted in 20 μL mixture comprising 4 μL of 5 × FastPfu buffer, 2 μL of 2.5 mM dNTPs, 0.8 μL of primers (5 μM), 1 U of TransStart Fastpfu DNA polymerase and 10 ng of template DNA. The PCR cycling was as follows: 95°C for 3 min; 29 cycles of denaturation at 95°C for 30 s, annealing at 55°C for 30 s, extension at 72°C for 45 s; final extension at 72°C for 10 min. The PCR amplification product was purified using the AxyPrep DNA Gel Extraction Kit (Axygen Biosciences, Union City, CA, United States), and the DNA was quantified using QuantiFluor^™^-ST (Promega, United States). The purified amplicons were pooled in equimolar and sequenced paired-end (2 × 300 bp) on the Illumina MiSeq platform (Illumina, San Diego, United States) according to the standard protocols. After subsampling each sample to an equal sequencing depth according to the minimum number (27,570) of sample sequences, operational taxonomic units (OTUs) were clustered using Usearch7.0 method of the QIIME1.9.1 with 97% similarity cutoff. The taxonomic assignment of each OTU representative 16S rRNA gene sequence was determined by Silva 128 16S rRNA database^1^ using confidence threshold of 70%.

### Statistical analysis

The alpha diversity indices including Shannon, Chao1 and Good’s coverage were calculated to measure the species richness and diversity of the community using Mothur (version 1.30.2) (Sun et al., 2016; Bullock et al., 2017). For beta diversity, non-metric multidimensional scaling analysis (NMDS) were performed with ANOSIM based on Bray-Curtis distance matrices using the “vegan” package (version 3.9) in R software (version 4.1.1). The differences in bacterial diversity and richness between different groups were analyzed by Wilcoxon signed-rank test. The relationship between environmental factors and bacterial community structure was evaluated by distance-based redundancy analysis (db-RDA) with 999 Monte Carlo permutation tests using the Canoco software (version 5.0, Microcomputer Power). The correlations between environmental factors and bacterial 16S rRNA gene abundances were conducted using Spearman correlation test. Mann–Whitney U test was used to detect the difference of total bacterial abundance between summer and winter. The difference between the FL and PA bacterial abundance was examined by Independent Samples *t*-test. These statistical analyses were performed on SPSS version 25.0 (SPSS, Chicago, IL, United States). In addition, Mantel test based on Pearson’s correlations was carried out by the “ggcor” package (version 0.9.4) in R (v4.2.1). The test for differences in microbial composition between groups was performed by the software STAMP (v2.1.3) (Parks et al., 2014). The map of sampling sites was created using Ocean Data View (ODV, v5.1.7) and figures were drawn by Origin 2022b software^2^ or GraphPad Prism 6.01.

A null model analysis was conducted following the statistical framework outlined by Stegen et al. (2013, 2015). The key metrics employed to discern these processes were the beta nearest taxon index (βNTI) and the Bray-Curtis-based Raup-Crick metric (RCbray), both calculated using the “picante” package (version 1.8.1) (Kembel et al., 2010). βNTI values exceeding 2 indicate that deterministic processes predominantly shape community structure. Specifically, βNTI values below −2 or above 2 correspond to homogeneous and heterogeneous selection, respectively. When |βNTI| values fall within the range of −2 to 2, it suggests that stochastic processes are the primary drivers of community assembly. These specific stochastic processes can be further deduced by considering the RCbray values. For |βNTI| < 2, RCbray < −0.95, RCbray > 0.95, and |RCbray| < 0.95 represent homogenizing dispersal, dispersal limitation, and ecological drift, respectively.

Co-occurrence networks were constructed using the “igraph” (version 0.10.7), “Hmisc” (version5.1–1) and “qvalue” (version 2.34.0) libraries in R (v4.2.1). To simplify the analysis, we retained only those Operational Taxonomic Units (OTUs) that had a relative abundance exceeding 0.01% across all samples and were present in more than 20% of the samples. We calculated pairwise Spearman’s correlations between these selected OTUs. Correlation coefficients exceeding |0.7|, with a *p*-value below 0.01 (adjusted using the Benjamini and Hochberg method), were considered as meaningful relationships. We assessed both network-level and node-level topological features of the co-occurrence network. Network-level characteristics included average degree, average network distance, average clustering coefficient, modularity index and diameter. Node-level characteristics encompassed nodes, edges, and proportion of positive correlation. Finally, the network visualization was done using Gephi (version 0.10.1). In each cooccurrence network, keystone species with high degree and low betweenness centrality were identified as those ranking within the top 20% for degree centrality among all nodes, and simultaneously ranking within the bottom 20% for betweenness centrality among nodes with high degree as described by Berry and Widder (2014).

### Data availability statement

Raw reads from the 16S rRNA gene amplicon sequencing were deposited in the NCBI BioProject database under the accession number PRJNA648032 (summer samples from June 2018) and PRJNA985391 (winter samples from December 2019).

## Results

### Hydrological conditions and environmental gradients

**Figure 2:**
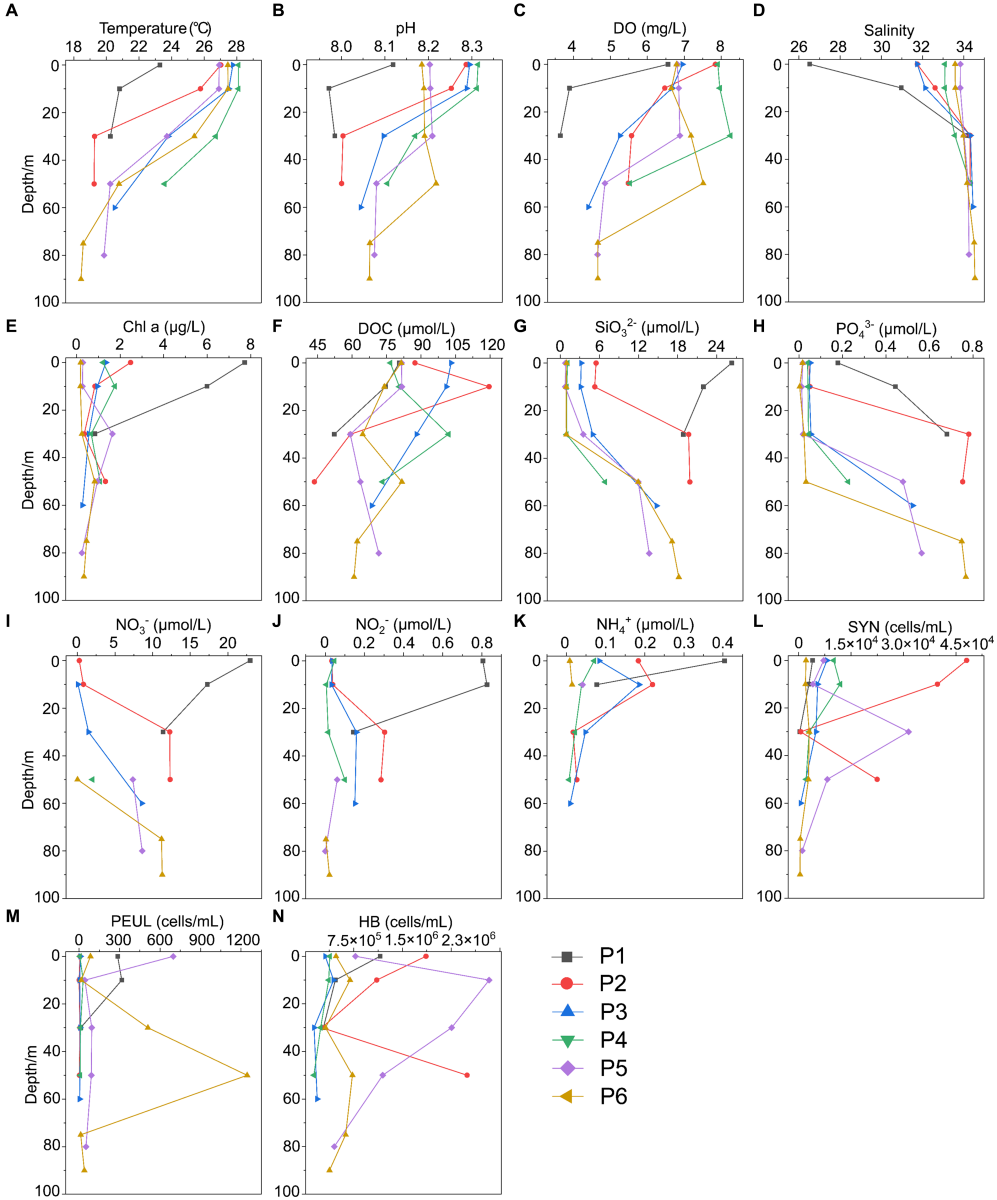
Environmental parameters along the PN section in summer. **(A)** Temperature; **(B)** pH; **(C)** DO; **(D)** Salinity; **(E)** Chl a, Chlorophyll a; **(F)** DOC; **(G)** SiO32-; **(H)** PO43-; **(I)** NO3-; **(J)** NO2-; **(K)** NH4+; **(L)** SYN, Synechococcus; **(M)** PEUL, picoeukaryotes; **(N)** HB, heterotrophic bacteria.

In summer, surface seawater from P1 exhibited low temperature (23.28°C) and salinity (26.51 PSU) compared to other sites, indicating a strong influence by the river plume of Changjiang Diluted Water (CDW). Correspondingly, the dissolved inorganic nitrogen (NO_3_^−^, NO_2_^−^, NH_4_^+^) and silicate (SiO_3_^2−^) showed highest concentrations, while phosphate (PO_4_^3−^) was depleted in P1 surface water (Figure 2; Supplementary Table S1). The temperature (25.7–27.7°C) and salinity (31.7–32.6) of P2 and P3 surface seawater suggested that these samples were also located in the water mass of continental coastal water (CCW) (Zhou et al., 2019). In comparison, the surface seawater of P4, P5 and P6 seemed to be influenced by the Taiwan Warm Current (TWC), a major current from the open sea with its surface water originating from the Taiwan Strait (Shi et al., 2014), resulting in a salinity of ~34. Most importantly, clear features of the Kuroshio subsurface water (KSSW) were observed in the subsurface water along the PN section, representing by distinctive temperature of as low as 18°C and high salinity of over 34 PSU (Yang et al., 2018). The invasive KSSW water that was rich in nutrients resulted in increased SiO_3_^2−^ and PO_4_^3−^ concentrations in the subsurface seawater of P2-P6 below 30 m (Figure 2; Supplementary Table S1).

**Figure 3:**
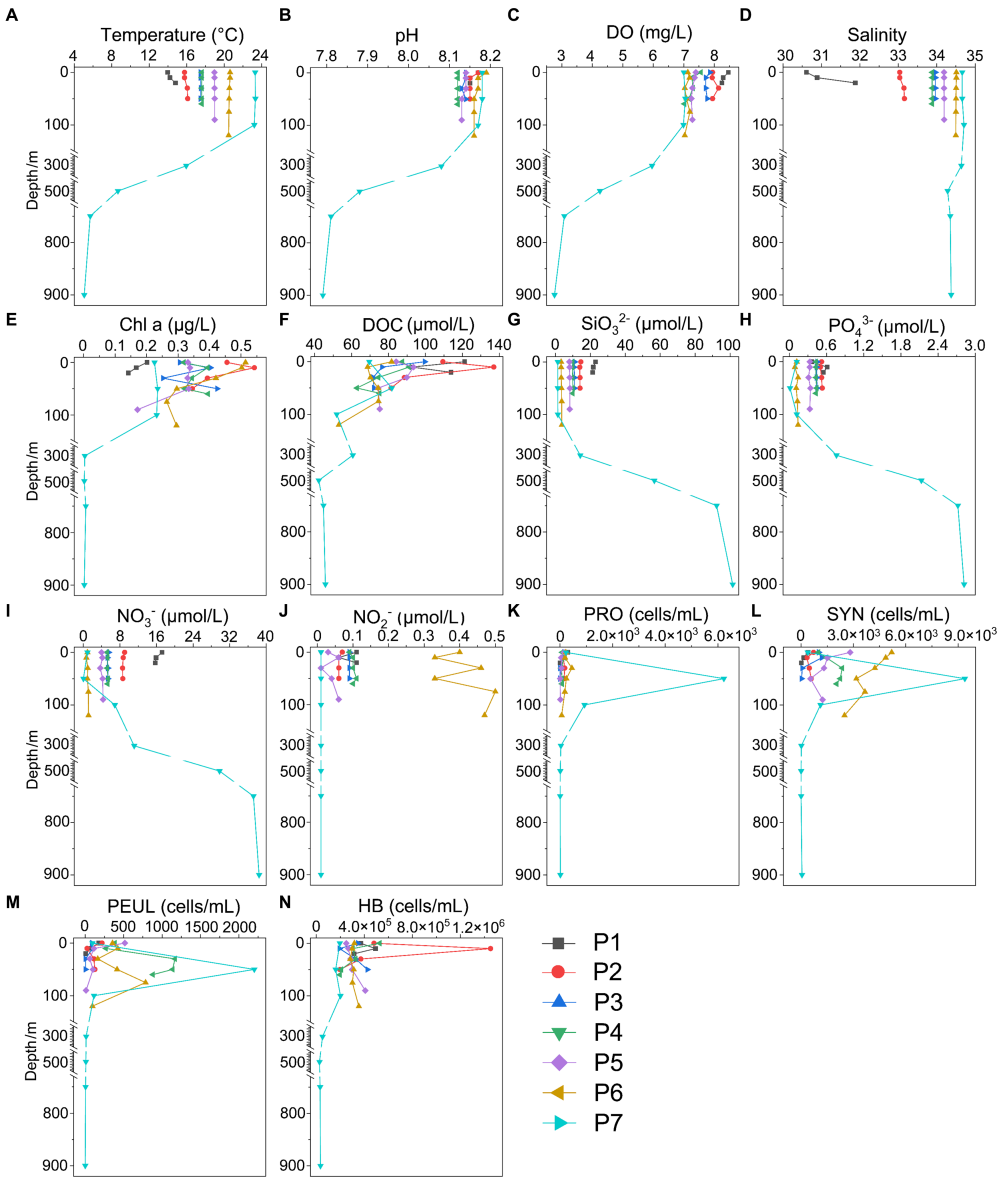
Environmental parameters along the PN section in winter. **(A)** Temperature; **(B)** pH; **(C)** DO; **(D)** Salinity; **(E)** Chl a, Chlorophyll a; **(F)** DOC; **(G)** SiO32-; **(H)** PO43-; **(I)** NO3-; **(J)** NO2-; **(K)** NH4+; **(L)** SYN, Synechococcus; **(M)** PEUL, picoeukaryotes; **(N)** HB, heterotrophic bacteria.

The hydrological conditions along PN section during winter were markedly different from those in summer (Figure 1). The reduction in Changjiang River discharge and the prevailing northeasterly wind in winter confined the influence of the Changjiang River water to a narrow band along the coast. Consistent with Yang et al. (2018), the seawater along PN section was well mixed from bottom to top due to the strong northeast monsoon (Figure 3; Supplementary Table S2). Although clear vertical variations of environmental parameters such as the temperature and salinity along water depths were observed in summer, the temperature, salinity, pH, DO and nutrients were more homogeneous above 100 m in P2-P7 during winter with no evident vertical variations. However, the typical KSSW was largely absent in winter (Yang et al., 2018), while Kuroshio surface water (KSW) and also TWC intruded along the PN section, resulting in the high salinity in P3-P7. Generally, the concentrations of NO_3_^−^, SiO_3_^2−^, and PO_4_^3−^ were higher in the surface seawater samples of winter compared to those of summer (*p* < 0.05, Wilcoxon rank sum test), as indicated by previous studies (Wang et al., 2003; Zhang et al., 2007). The temperature, salinity and nutrient gradients along PN section indicated that these samples reflected typical hydrological conditions and environmental gradients in both summer and winter (Figure 2, 3; Supplementary Tables S1, S2). Distinct variations of other environmental factors between summer and winter seawater samples were also observed. In summer, the Chl *a* content was highest in P1 surface seawater under the influence of Changjiang River plume (7.7 μg/L), representing a location with high primary productivity, and the invasion of TCW and Kuroshio water led to lower Chl *a* concentration in P2-P7. Seawater from P1 also exhibited the lowest DO concentrations in summer. On the contrary, in winter, the Chl *a* content was lowest in P1 (0.14–0.20 μg/L), and was consistently higher in P2-P7 (0.17–0.54 μg/L) above 100 m. The DO content of P1 was the highest and reduced from P2-P7 in winter (Figure 3).

### Variations in microbial abundance and diversity

**Figure 4:**
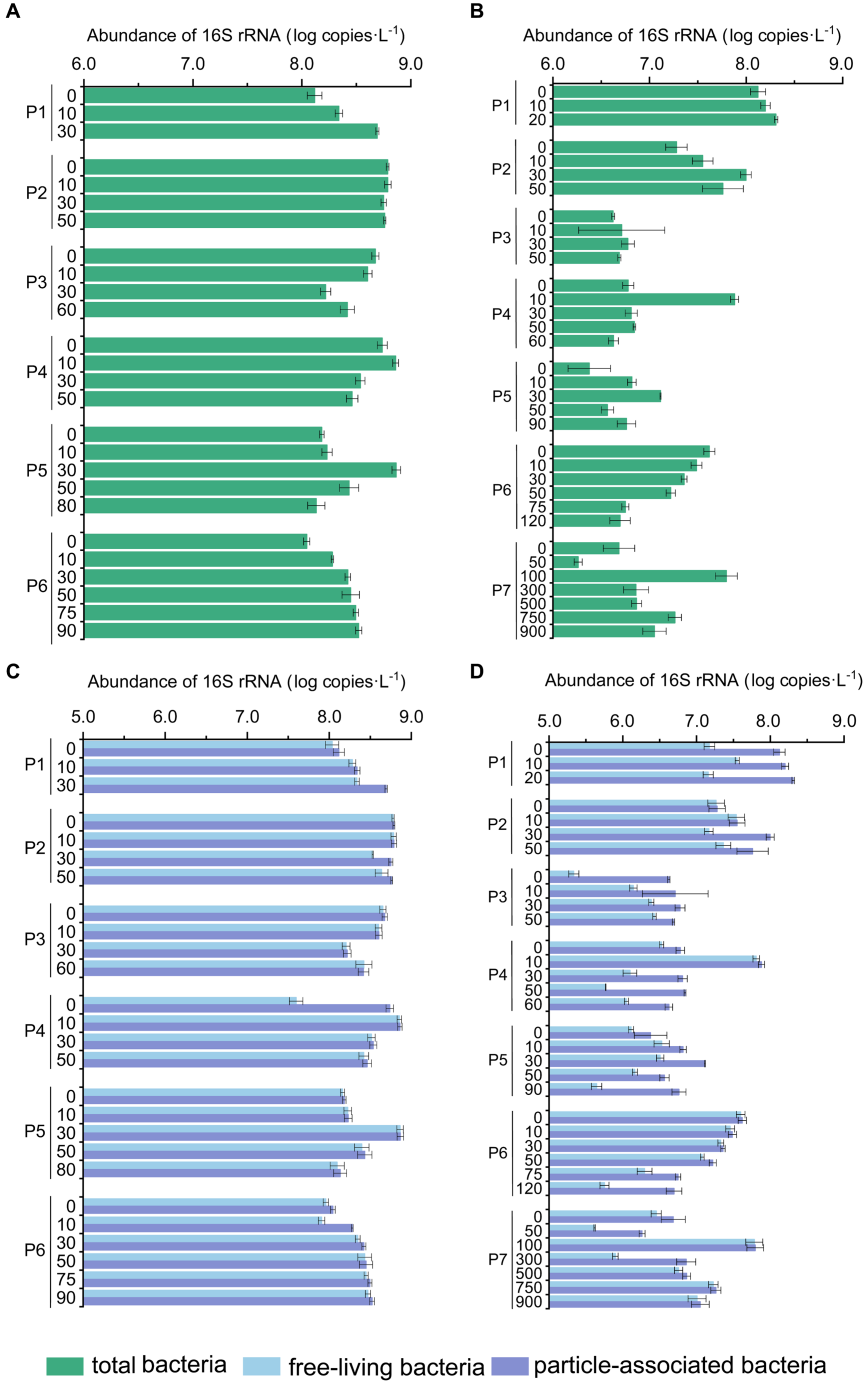
The abundance of bacterial 16S rRNA genes in the PN section during summer and winter. **(A)** Total bacterial 16S rRNA gene abundance in summer; **(B)** total abundance of bacterial 16S rRNA gene in winter; **(C)** free-living and particle-associated bacterial 16S rRNA gene abundance in summer; **(D)** free-living and particle-associated bacterial 16S rRNA gene abundance in winter.

As shown by the flow cytometry results, the average counting of heterotrophic bacteria in summer was 7.92 ± 7.56 × 10^8^ cells L^−1^ and 3.12 ± 2.37 × 10^8^ cells L^−1^ in winter. The total microbial abundance along the PN section was also higher in summer (1.11 × 10^8^ copies L^−1^ – 7.45 × 10^8^ copies L^−1^) than those in winter (1.83 × 10^6^ copies L^−1^ – 1.59 × 10^8^ copies L^−1^) (*p* < 0.01, Mann–Whitney U test) (Figure 4). The reduction in total microbial abundance was more evident in winter samples from P3-P7 (1.47 ± 0.23 × 10^7^ copies L^−1^) when compared with the summer samples from P3-P6 (3.28 ± 0.35 × 10^8^ copies L^−1^) (*p* < 0.01, Mann–Whitney U test), indicating that less microbes were brought in to the ESC shelf along PN section by Kuroshio current in winter. Additionally, in winter, the microbial abundance of P3-P7 (1.83 × 10^6^ copies L^−1^ – 7.61 × 10^7^ copies L^−1^) was significantly lower compared to those of P1-P2 (3.62 × 10^7^ copies L^−1^ – 2.04 × 10^8^ copies L^−1^) (*p* < 0.01, Mann–Whitney U test). Vertically, the microbial abundance of P1 exhibited an increasing trend with water depth in both summer and winter; no apparent variation pattern with the water depth was observed in other PN sites. When considering the different microbial lifestyles, the abundance of FL bacteria (3.00 ± 0.30 × 10^8^ copies L^−1^) was generally higher than that of PA (6.15 ± 0.73 × 10^7^ copies L^−1^) in summer (*p* < 0.05, Independent Samples *t*-test), with exceptions in some specific samples (P1_30m, P4_0m). While in winter, the abundance of PA bacteria (2.01 ± 0.31 × 10^7^ copies L^−1^) was slightly higher than that of FL (1.32 ± 0.22 × 10^7^ copies L^−1^) (*p* < 0.05, Independent Samples *t*-test), with exceptions in pelagic regions (P6-P7).

**Figure 5:**
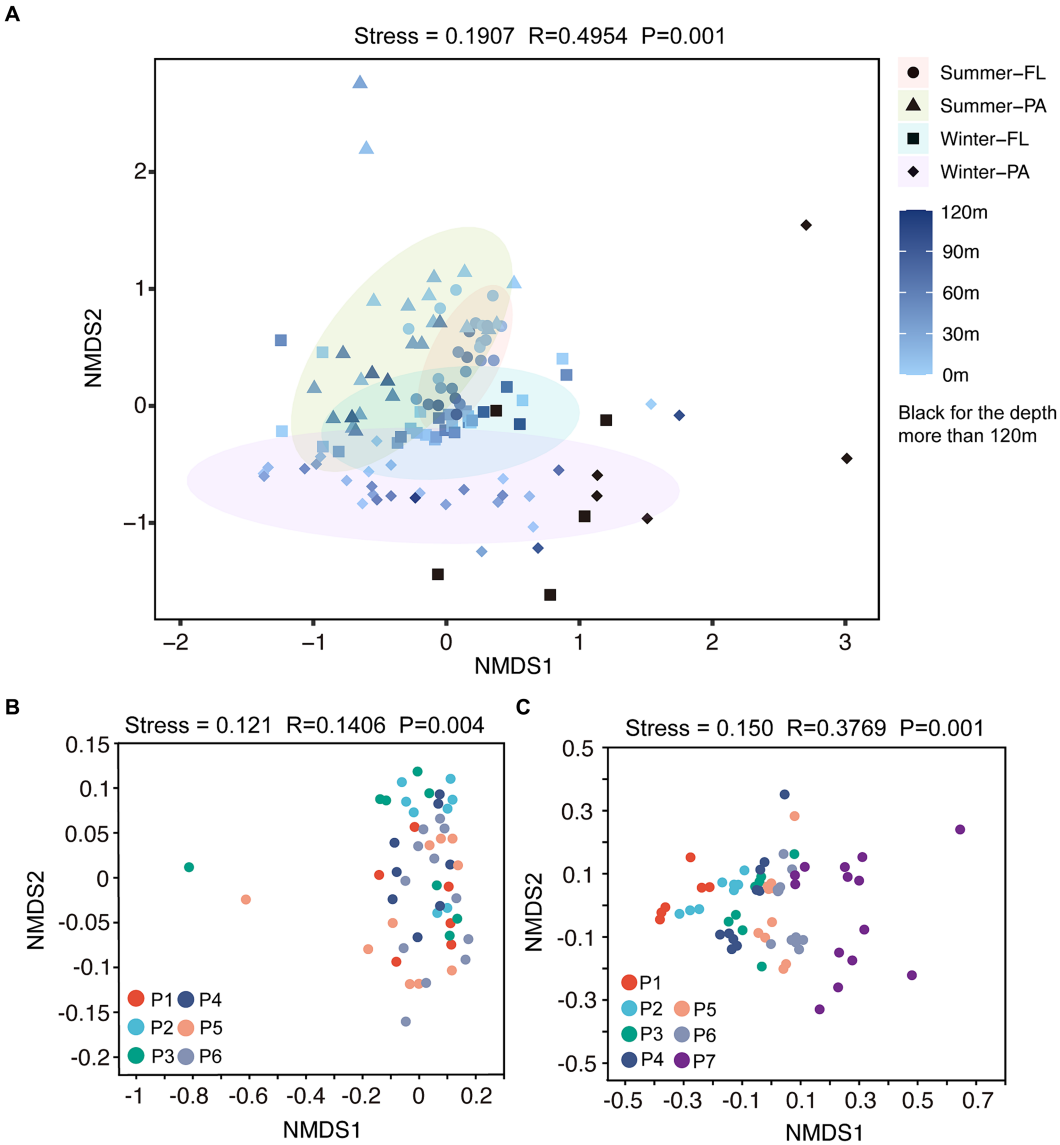
NMDS analysis on OTU level along the PN section. FL, Free-living; PA, Particle-associated. **(A)** NMDS analysis of all samples from summer and winter; **(B)** NMDS analysis of summer samples; **(C)** NMDS analysis of winter samples.

A total of 59,594±15,349 reads in each sample were obtained and were clustered into 12,015 OTUs at a 97% similarity level. The diversity and richness of microbial communities along the PN section indicating by the Shannon and Chao 1 indices, respectively, presented distinct variation patterns between summer and winter (Supplementary Figure S1; Supplementary Tables S3, S4). In summer, the Shannon and Chao 1 indices remained at relatively stable levels along the PN section (*p* > 0.05, Wilcoxon signed-rank test); both Shannon and Chao 1 were slightly higher in PA communities when compared to the FL counterparts. While in winter, the Chao 1 index showed a clear decreasing trend from P1 to P7 in both PA and FL communities (*p* < 0.05, Wilcoxon signed-rank test, Supplementary Figures S1C,D); the Shannon index also decreased significantly from P4-P7, especially in the PA communities of these offshore samples (*p* < 0.05, Wilcoxon signed-rank test). The community distribution pattern revealed by Nonmetric multidimensional scaling (NMDS) analysis showed a shift of microbial communities in different seasons (Figure 5A), with separations of FL and PA samples in both summer (*p* < 0.001, Mann–Whitney U test) and winter (*p* < 0.05, Mann–Whitney U test). Additionally, the winter communities presented a more evident trend of spatial shift along the PN section (from P1-P7, Figure 5C) compared with the summer communities (Figure 5B).

### Microbial community compositions along the PN section in summer and winter

**Figure 6:**
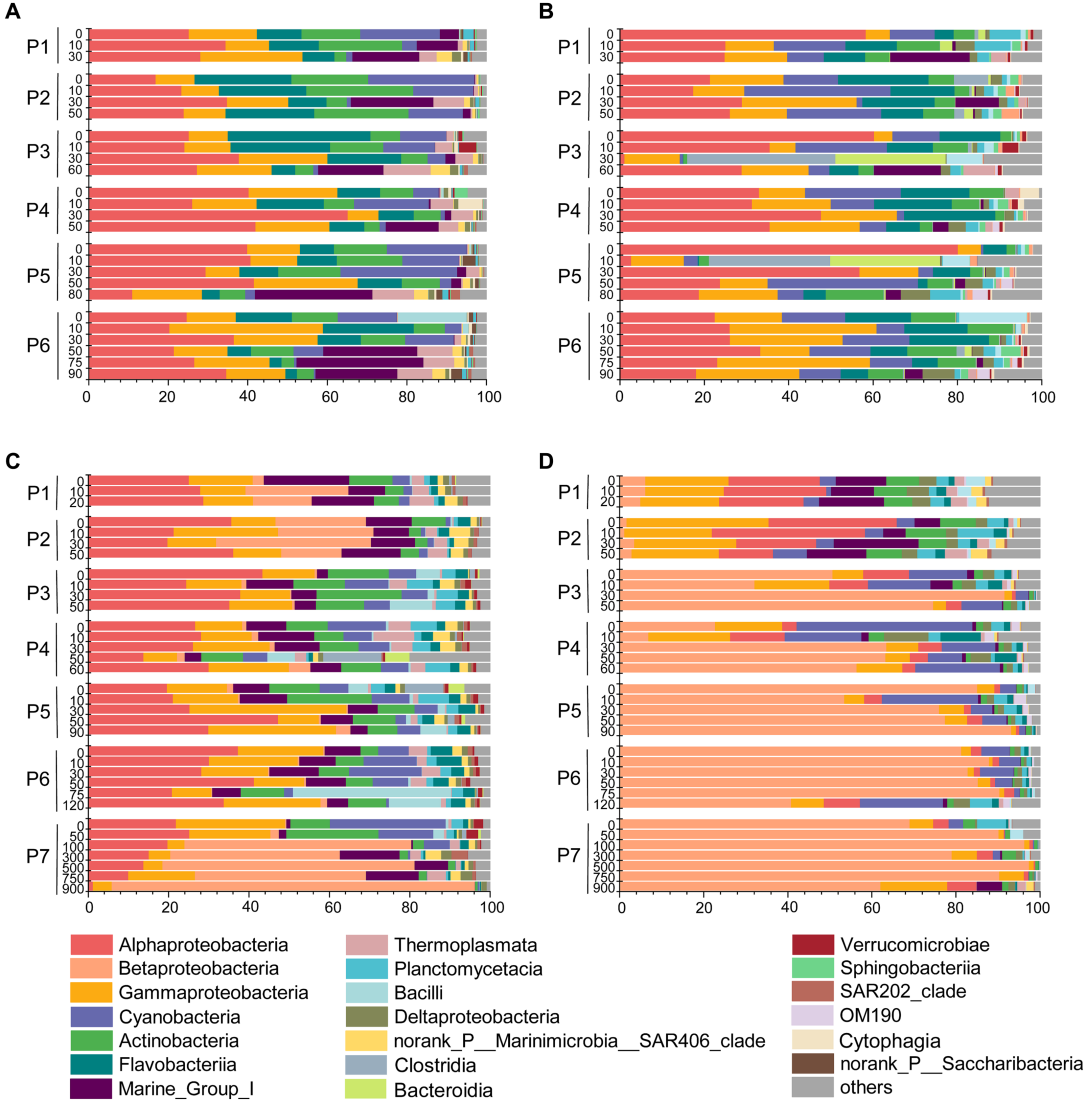
Microbial communities along the PN section during summer and winter (in class level). **(A)** Free-living communities in summer; **(B)** particle-associated communities in summer; **(C)** Free-living communities in winter; **(D)** particle-associated communities in winter.

**Figure 7:**
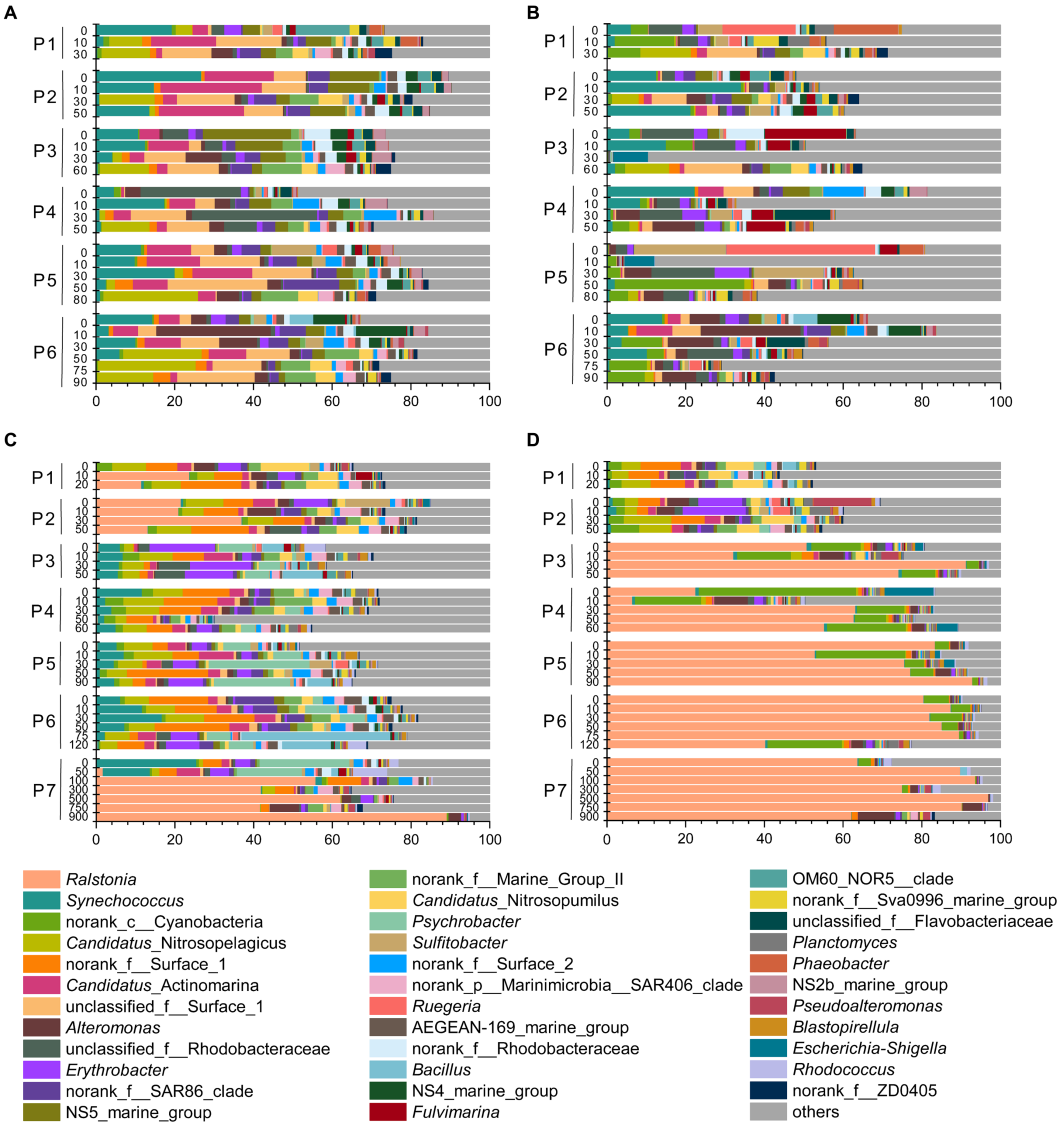
Microbial communities along the PN section during summer and winter (in genus level). **(A)** Free-living communities in summer; **(B)** particle-associated communities in summer; **(C)** free-living communities in winter; **(D)** particle-associated communities in winter.

Based on the distinct seasonal patterns of microbial abundance and diversity shown above, we further investigated the dominant microbial communities along the PN section in summer and winter. In summer, the dominant microbial classes were similar in FL and PA fractions, including *Alphaproteobacteria* (30.94% in FL and 30.89% in PA), *Gammaproteobacteria* (16.34% in FL and 16.36% in PA) and *Flavobacteria* (13.12% in FL and 10.65% in PA), *Cyanobacteria* (9.54% in FL and 11.81% in PA) and *Actinobacteria* (10.84% in FL and 5.97% in PA) (Figures 6A,B). However, more apparent differences were shown at the genus level between summer FL and PA communities (Figure 7; Supplementary Figure S3C). In the FL samples, genera belonging to SAR11 clade, *Actinobacteria* and Marine Group I were most abundant, and *Candidatus* Nitrosopelagicus in *Thaumarchaeota* showed high abundance in several subsurface water samples (30-80 m) (Figure 7A; Supplementary Figure S3C). While in the PA samples (Figure 7B), *Synechococcus* (6.53%), *Alteromonas* (4.04%), *Sulfitobacter* (3.03%) and unidentified genera in *Cyanobacteria* (4.92%) and *Rhodobacteraceae* (4.54%) were highly abundant. Consistent with the NMDS results (Figure 5B), the dominant microbial communities showed no evident differences from nearshore and offshore along the PN section in summer.

In winter, the microbial community composition along the PN section was featured by a dominance of bacteria belonging to *Ralstonia* (*Betaproteobacteria*), which accounted for 12.49% in FL and 56.69% in PA samples (Figures 7C,D; Supplementary Figure S3D). In the FL fractions, these *Ralstonia* mainly exist in P1 (11.75%), P2 (23.18%), and P7 (41.82%) with the abundance increased with water depth. While in the PA fractions, the abundance of *Ralstonia* slightly increased from P3 (62.14%) to P7 (81.67%), reaching as high as 90.28% in P7_750m. Except *Betaproteobacteria* (mostly *Ralstonia*), *Alphaproteobacteria* (26.54% in FL and 7.80% in PA) and *Gammaproteobacteria* (15.54% in FL and 9.44% in PA) were the abundant groups in winter samples as in summer (Figures 6C,D, 7C,D). The abundance of *Flavobacteria*, *Actinobacteria* and *Cyanobacteria* were lower in winter compared with those in summer (Supplementary Figure S2A).

### Relationships between environmental variables and microbial bacterial community structure

**Figure 8:**
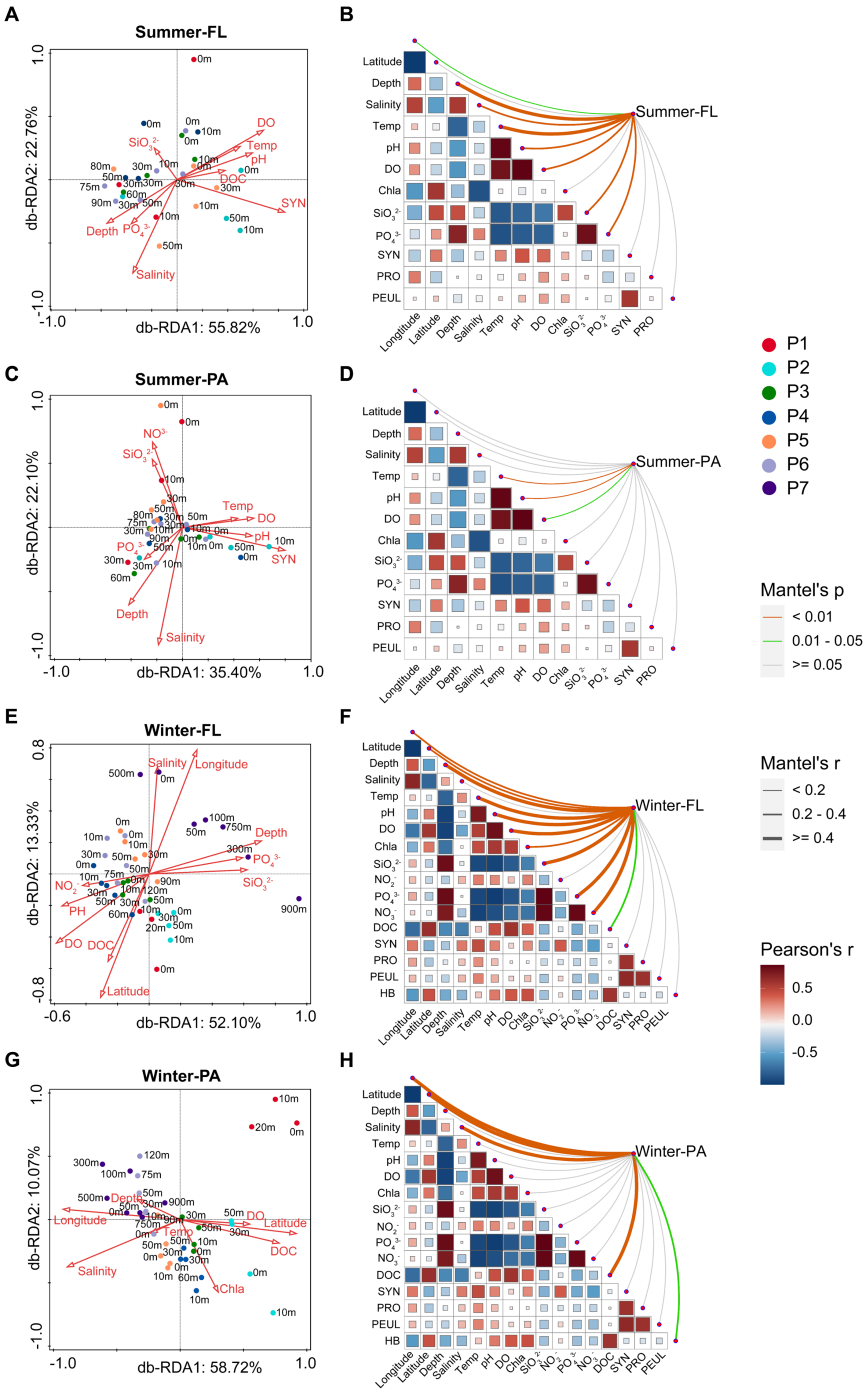
db-RDA analysis and Mantel test showing the relationship between microbial community structure based on OTU level and environmental factors along the PN section during summer and winter. FL: Free-living; PA: Particle-associated. **(A,B)** Free-living samples in summer; **(C,D)** particle-associated samples in summer; **(E,F)** free-living samples in winter; **(G,H)** particle-associated samples in winter. In **(B,D,F,H)**, the size and color of the squares indicate the correlation coefficients between the environmental factors, the thickness of the lines indicate the correlation of the Mantel test between the microbial community and the environmental factors, and the color of the lines indicates the significance of the Mantel test between the microbial community and the environmental factors.

db-RDA based on Bray-Curtis distances was used to investigate the influence of environmental parameters on microbial community compositions along the PN section (Figure 8). In general, depth, salinity, temperature, DOC, pH, DO, pH, SiO_3_^2−^, PO_4_^3−^ NO_3_^−^, and SYN were correlated with the distribution of microbial communities along the PN section in summer. However, except for the above environmental parameters, latitude and longitude also significantly affected the winter microbial communities, while SYN showed no effect.

The Mantel test results indicated that stronger correlations between various environmental factors and winter communities than summer communities along the PN section (Figure 8; Supplementary Table S5). The changes of summer FL communities were significantly correlated with temperature, depth and, SiO_3_^2−^ (*r* > 0.4, *p* < 0.001), and weakly correlated with salinity, pH, DO, and PO_4_^3−^ (*r* > 0.2, *p* < 0.001). However, only temperature and pH showed weak correlations with the summer PA communities (*r* < 0.2, *p* < 0.001). In winter, the FL communities were significantly correlated with depth, temperature, DO, PO_4_^3−^, SiO_3_^2−^, NO_3_^−^, pH, longitude and latitude (*r* > 0.2, *p* < 0.001), whereas salinity, longitude, latitude and DOC were significantly correlated with the PA communities (*r* > 0.4, *p* < 0.001).

The Spearman correlations between the bacterial abundance and the environmental parameters were calculated (Supplementary Table S6). In summer, the abundance of both total (*r =* 0.491, *p* < 0.05) and FL bacteria (*r =* 0.426, *p* < 0.05) showed significant positive correlation with Chl *a*. The abundance of summer PA bacteria was positively correlated with PO_4_^3−^ (*r =* 0.404, *p* < 0.05) and NO_3_^−^ (*r =* 0.708, *p* < 0.01) and negatively correlated with pH (*r =* −0.460, *p* < 0.05) and DOC (*r =* −0.636, *p* < 0.01). In winter, the abundance of total bacteria was positively correlated with NO_3_^−^ (*r =* 0.401, *p* < 0.05). In particular, the abundance of PA bacteria showed significant positive correlation with latitude (*r =* 0.491, *p* < 0.01) and DO (*r =* 0.457, *p* < 0.01), and negatively correlated with longitude (*r =* −0.491, *p* < 0.01) and salinity (*r =* −0.457, *p* < 0.01). In addition, we found the dominant *Ralstonia* in winter PA samples was positively correlated with longitude, depth, salinity and temperature, while chemical parameters such as DO, SiO_3_^2−^, PO_4_^3−^, NO_3_^−^, and DOC were found to be negatively correlated with *Ralstonia* (Supplementary Table S7).

### Assembly process structuring the microbial communities in the PN section

**Figure 9:**
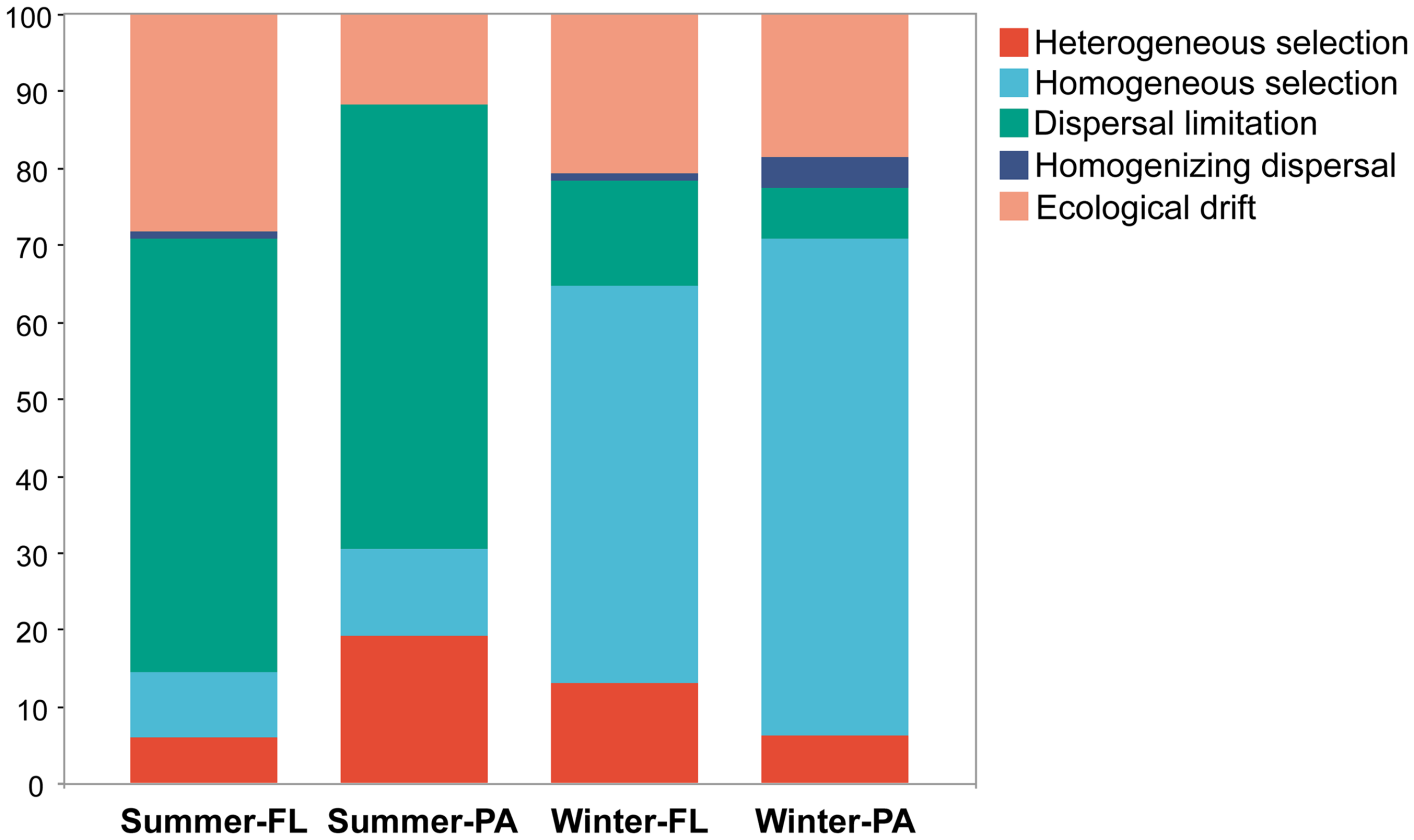
The assembly processes of different communities with the null model. FL, Free-living; PA, Particle-associated.

The βNTI analyses based on the null model revealed a clear difference of dominant processes in structuring the microbial communities along the PN section between winter and summer (Figure 9). In summer, the stochastic process represented by dispersal limitation was the most important process in either FL and PA communities (56.3 and 57.8%, respectively), while in winter, the major process turned out to be the deterministic process, homogeneous selection (51.5% in FL and 64.5% in PA). Additionally, heterogeneous selection accounted for higher proportions in determining PA communities (5.8%) compared to the FL communities (1.9%) in summer. Although selection was the most important structuring process in winter, the contribution of dispersal limitation was higher for the FL (13.7%), whereas homogenizing dispersal was slightly higher for the PA (3.9%) among the stochastic processes.

### Microbial community associations along the PN section

**Figure 10:**
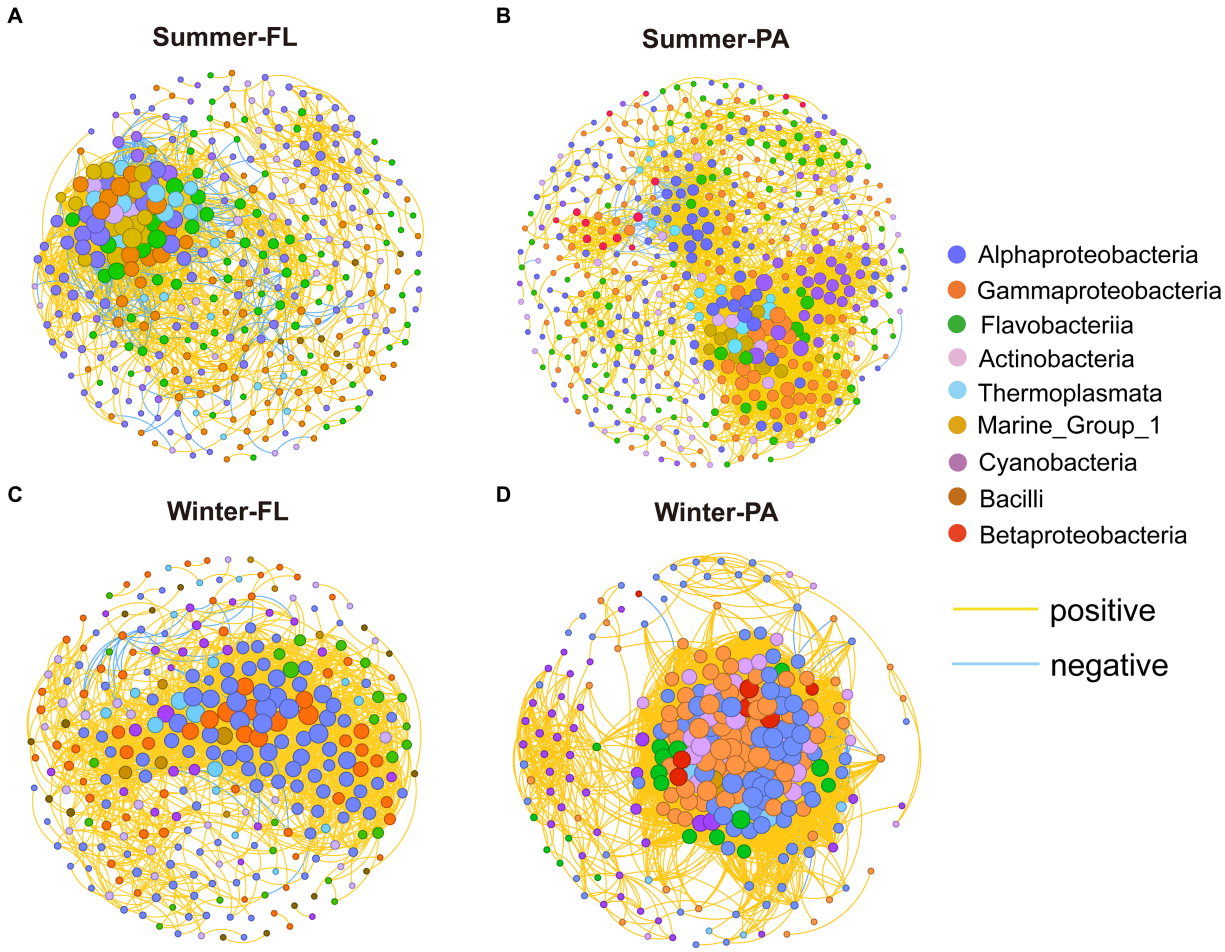
Co-occurrence networks of the microbial community from different season and lifestyle. Each connection shown has a correlation coefficient > |0.7| and a *p* value < 0.01. The size of each node is proportional to the number of connections. The OTUs were colored by subcommunity and taxonomy, respectively. FL, Free-living; PA, Particle-associated. **(A)** Co-occurrence networks of summer Free-living community; **(B)** co-occurrence networks of summer Particle-associated community; **(C)** co-occurrence networks of winter Free-living community; **(D)** co-occurrence networks of winter Particle-associated community.

To study the microbial co-occurrence patterns along the PN section, we built co-occurrence networks for samples from different seasons and different lifestyles (Figure 10). Generally, the summer network exhibited lower average degree and average clustering coefficient compared with those of the winter network, whereas the average network distance and modularity was higher in summer than in winter. These results suggested that the microbial communities tended to be more connected in winter than in summer along the PN section. Specifically, the network of winter PA communities presented distinct topological properties compared with either summer communities or winter FL counterparts, showing lower node numbers and extremely high edge numbers (Supplementary Table S9). Correspondingly, higher average degree and clustering coefficient was shown by the winter PA network, while its modularity was lower (0.099). The OTUs from *Alphaproteobacteria*, *Gammaproteobacteria*, *Flavobacteria*, *Actinobacteria*, and *Cyanobacteria* displayed more correlations with others in these networks (Figure 10; Supplementary Table S9). Keystone OTUs in each network with high degree (>25) and low betweenness centrality (<262.32) were identified. Interestingly, the keystone OTUs in winter were dominated by *Alpha-* and *Gammaproteobacteria* (100% for FL and 89% for PA), while high proportions of archaeal keystone OTUs (*Thaumarchaeota* and *Euryarchaeota*) were identified in summer microbial networks (43% for FL and 28% for PA) (Supplementary Table S8).

We further built separate co-occurrence networks for nearshore and offshore PN section samples, respectively, which were influenced by the distinct hydrographic conditions (Supplementary Figure S4). In both seasons, the networks of offshore FL samples possessed more edges and higher average degree than the nearshore FL samples, while the networks of offshore PA samples were with less edges and lower average degree than the nearshore samples (Supplementary Table S10). This indicated that the FL microbial networks were more complex in offshore sites, whereas the nearshore PA microbial networks were more complex in nearshore sites along the PN section. Additionally, the complexity of the winter PA microbial network at P5-P7 was significantly reduced, with less nodes, edges and lower average degree, possibly due to the high abundance of *Ralstonia* species. In the networks of winter samples, we found these *Ralstonia* exhibited mainly positive correlations with some *Alphaproteobacteria* and *Gammaproteobacteria* in P1-P2, while more negative correlations between *Ralstonia* and specific *Alphaproteobacteria*, *Gammaproteobacteria* and *Cyanobacteria* were found in P5-P7 (Supplementary Table S11), indicating that the dominance of *Ralstonia* may inhibit these bacteria communities.

## Discussion

Overall, we observed significant differences in hydrological conditions between winter and summer along the PN transect. In summer, the invasion of Kuroshio subsurface water (KSSW) into the subsurface seawater of P2-P6 below 30 m was evident, significantly influencing the environmental parameters along the PN section and resulting in vertical variations of temperature, salinity and nutrients (Figure 2; Supplementary Table S1) (Hur et al., 2000; Yang et al., 2012; Zhang Q. L. et al., 2014). While in winter, the vertical variations of these environmental parameters were homogenized by the strong mixture of bottom to top water caused by the northeast monsoon (Liu et al., 2021). Whether in summer or winter, the variations in environmental factors along the PN transect were jointly caused by the Changjiang Diluted Water (CDW) and the Kuroshio Current, with P1 and P2 exhibiting clear features of CDW (Figure 2, 3; Supplementary Tables S1, S2). With different characteristics in temperature, salinity and nutrients, the interactions between CDW and Kuroshio intrusion lead to complex chemical conditions, as well as the distribution and dynamics of microbial communities (Guo et al., 2012; Zhou et al., 2019).

Microbial community abundance, diversity, and taxa exhibited noticeable seasonal differences along the PN section, with more pronounced changes along the PN section in winter compared to those in summer. The total bacterial abundance decreased significantly from P1-P2 to P3-P7 (Figure 4), and the α-diversity indices exhibited a clear decreasing trend along PN section in winter (Supplementary Figures S1C,D). Additionally, the winter microbial communities exhibited a more distinct separation by sites along the PN section compared to the summer communities (Figure 5). Indeed, the db-RDA analysis and Mantel test revealed that latitude and longitude significantly affected the winter microbial communities but not the summer communities (Figure 8). These results indicate that the intrusion of KSSW may reduce the differences in microbial community among water layers and increase the lateral mixing along PN section during summer. However, in winter, despite enhanced vertical-horizontal mixing, the lateral variations in environmental gradients and hydrological conditions along the PN stations make the differences in microbial communities between sites more pronounced.

As indicated by previous studies and our findings on these ESC samples, FL and PA microorganisms can be different in their diversity, community and the driving environmental parameters (D'Ambrosio et al., 2014; Leu et al., 2022). This might be due to significantly higher carbon and nutrient levels in the particles compared with the surrounding seawater (Azam and Malfatti, 2007). Additionally, along the PN section, the source and chemical constitution of these particles could be largely influenced by the complex hydrological conditions. Most importantly, we found that the winter microbial communities along PN section, especially in the PA fractions, were characterized by the dominance of an unexpected group, *Ralstonia*. As an ubiquitous microorganism found in water and soil (Gilligan et al., 2003), a high abundance of *Ralstonia* in distinct seawater environment with unknown reason has been rarely reported. Recurrent ‘blooms’ of *Ralstonia* were reported the ultra-oligotrophic eastern Mediterranean, which is consistent with our finding that it occurs mainly in PA groups (Rosenberg et al., 2021). Additionally, the abrupt increase of *Ralstonia* was recently observed during February to June in a high-resolution study over 60 consecutive weeks at three coastal sites of China (Zhang et al., 2024), with its highest abundance comprised up to 90% of the PA community. *Ralstonia* has also been reported to be dominated in the water column and coral mucus in the reef habitats by Kegler et al. (2017). One possible explanation for the high abundance of *Ralstonia* along PN section in winter is that these *Ralstonia* might be brought to and spread along the PN section by the Kuroshio surface water and Taiwan Warm Current (TWC) that both flowed through Taiwan.

Based on the distinct hydrological conditions and microbial communities, we further explored the dominant ecological processes driving the microbial community assembly. In summer, stochastic processes made a major contribution to the microbial community assembly along the PN section, and this was consistent with that reported by Lian et al. (2023) from nearshore to offshore areas in the ECS. The complex hydrographic movements may be the main reason that allow for increased dispersal limitations in summer (Tsai et al., 2010). However, in winter, a dominance of deterministic process occurred. This was supported by the blooms of *Ralstonia*, which may exert selective effects on the overall community and result in a dominant role of deterministic processes (Xu et al., 2020; Zhang et al., 2024).

Additionally, microorganisms usually do not exist independently, but build complex networks of interactions. These interaction networks have a significant impact on the structure and diversity of microbial communities (Shiri et al., 2010; Lima-Mendez et al., 2015). The results of the co-occurrence network analysis for the different subgroups showed that most of the edges were positive, suggesting that overall microbial cooperation was more frequent than competition in the PN section. The topological properties of microbial co-occurrence network and the corresponding keystone species exhibit significant differences between winter and summer, especially in the network of winter PA community. Separate co-occurrence networks for nearshore and offshore PN section samples further indicated that the FL and PA communities showed different interaction patterns in nearshore and offshore sites. Additionally, the dominance of *Ralstonia* in P5-P7 during winter may influence the network structure. These results indicated that distinct hydrological conditions, environmental factors, and microbial community resulted in distinct interaction patterns among microorganisms in different seasons and lifestyles along the PN section.

Notably, OTUs instead of amplicon sequence variants (ASVs) were used in this study (Benjamin et al., 2017), which may reduce the sensitivity on detecting specific bacterial strains present in these PN samples and affect the accurate detection of α-diversity (Chiarello et al., 2022). Additionally, further analysis based on ASVs with finer resolution may lead to better discrimination on the diversity of these *Ralstonia* species, and help to provide more evidence on their source combined with future studies on terrestrial and seawater samples.

To summarize, the current study revealed distinct seasonal hydrological conditions in the ESC and the resulting differences in microbial ecology. Our results emphasized the importance of understanding microbial communities along with the complex water mixing in the ESC and revealed unexpected high abundance of terrestrial bacterial in winter. Moreover, this study also highlights the importance of conducting long-term and systematic investigations into the microbial communities in the ESC. Certain microbial taxa identified could serve as reliable indicator species that help to better understand the complex dynamics of water currents and masses in the ESC and surrounding areas.

## Conclusion

Through a comprehensive investigation of the microbial communities along the PN section during summer and winter, we showed clear temporal and spatial variations of microbial abundance, diversity and interaction patterns. These differences were closely related to the distinct hydrological conditions and the resulting environmental gradients along the PN section in winter and summer. Our results for the first time provided a glimpse on how hydrological conditions affect dynamic changes of microbial communities along the PN section, and provide new insights for the microbial assembly and interactions in this complex environment.

## Data availability statement

The datasets presented in this study can be found in online repositories. The names of the repository/repositories and accession number(s) can be found in the article/Supplementary material.

## Author contributions

JJ: Formal analysis, Investigation, Methodology, Visualization, Writing – original draft, Writing – review & editing. XL: Formal analysis, Investigation, Methodology, Visualization, Writing – original draft. WZ: Investigation, Methodology, Writing – review & editing. HS: Data curation, Investigation, Methodology, Writing – review & editing. ST: Investigation, Methodology, Writing – review & editing. X-HZ: Conceptualization, Funding acquisition, Investigation, Writing – review & editing. YZ: Conceptualization, Investigation, Writing – original draft, Writing – review & editing.

## Footnotes

^1^ http://www.arb-silva.de

^2^ https://www.originlab.com
