# Supplementary material for: Microbial community diversity from nearshore to offshore in the East China Sea
Source: Front Microbiol. 2024 May 27;15:1377001. doi: 10.3389/fmicb.2024.1377001 (PMC11166001; doi:10.3389/fmicb.2024.1377001)
Supplement: Supplementary file 1 [file Table_1.DOCX]

­­­­**Supplementary materials**

**Microbial community diversity from nearshore to offshore in the East China Sea**

**Jian Jin^1†^, Xiujie Liu^1†^, Wenbin Zhao^1^, Hao Sun^1^, Siyin Tan^1^, Xiao-Hua Zhang^1,2,3^ and Yunhui Zhang^2*^**

^1^College of Marine Life Sciences, and Frontiers Science Center for Deep Ocean Multispheres and Earth System, Ocean University of China, 5 Yushan Road, Qingdao 266003, China

^2^ Key Laboratory of Evolution & Marine Biodiversity (Ministry of Education) and Institute of Evolution & Marine Biodiversity, Ocean University of China, Qingdao 266003, China

^3^Laboratory for Marine Ecology and Environmental Science, Laoshan Laboratory, Qingdao 266071, China

***Correspondence:**

Yunhui Zhang, zhangyunhui@ouc.edu.cn


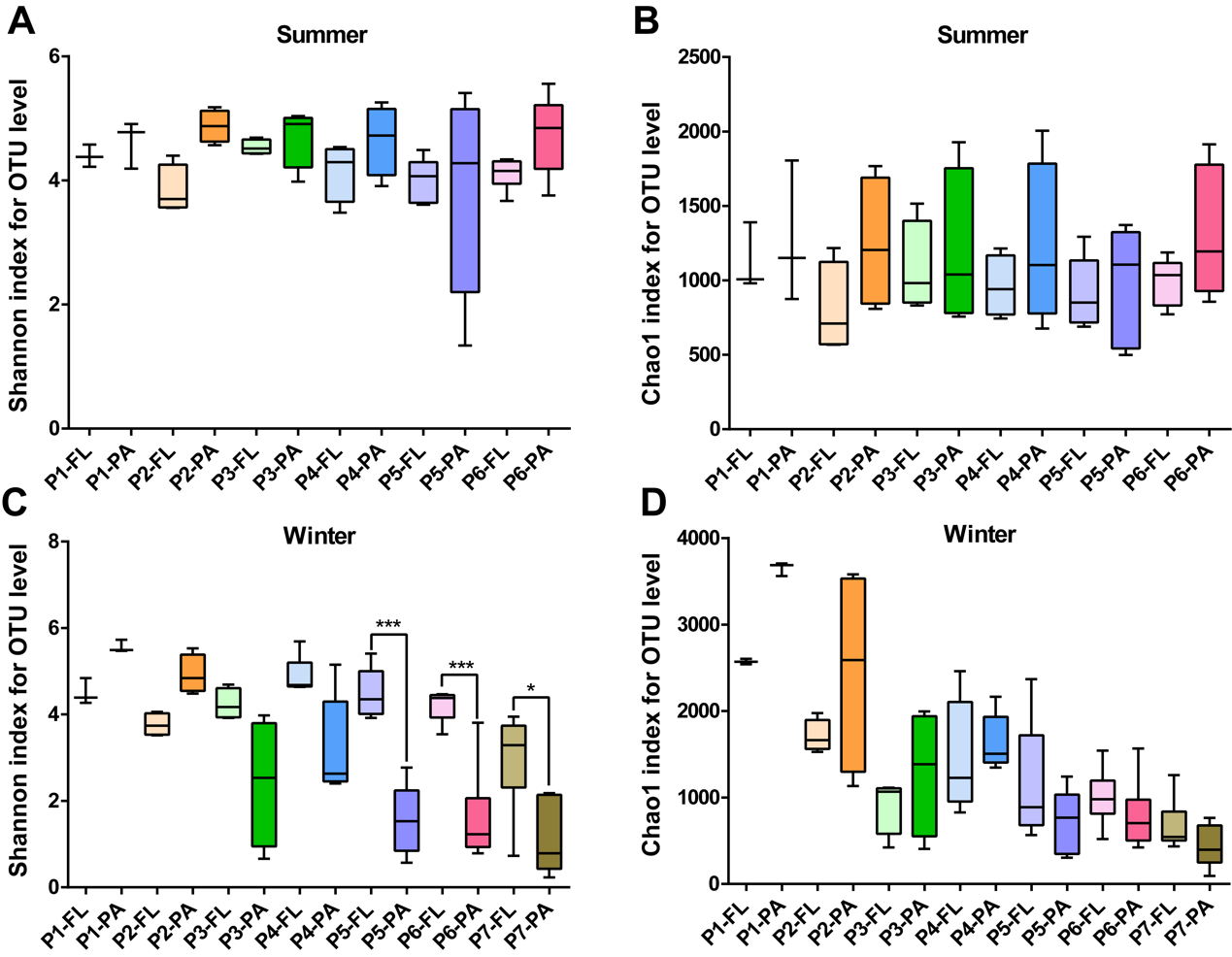


**Fig.S1** Comparison of diversity and richness of free-living and particle-associated microbial community structure along the PN section during summer and winter. FL: Free-living; PA: Particle-associated. **(A)** and **(B)**, Shannon index and Chao 1 index in summer; **(C)** and **(D)**, Shannon index and Chao 1 index in


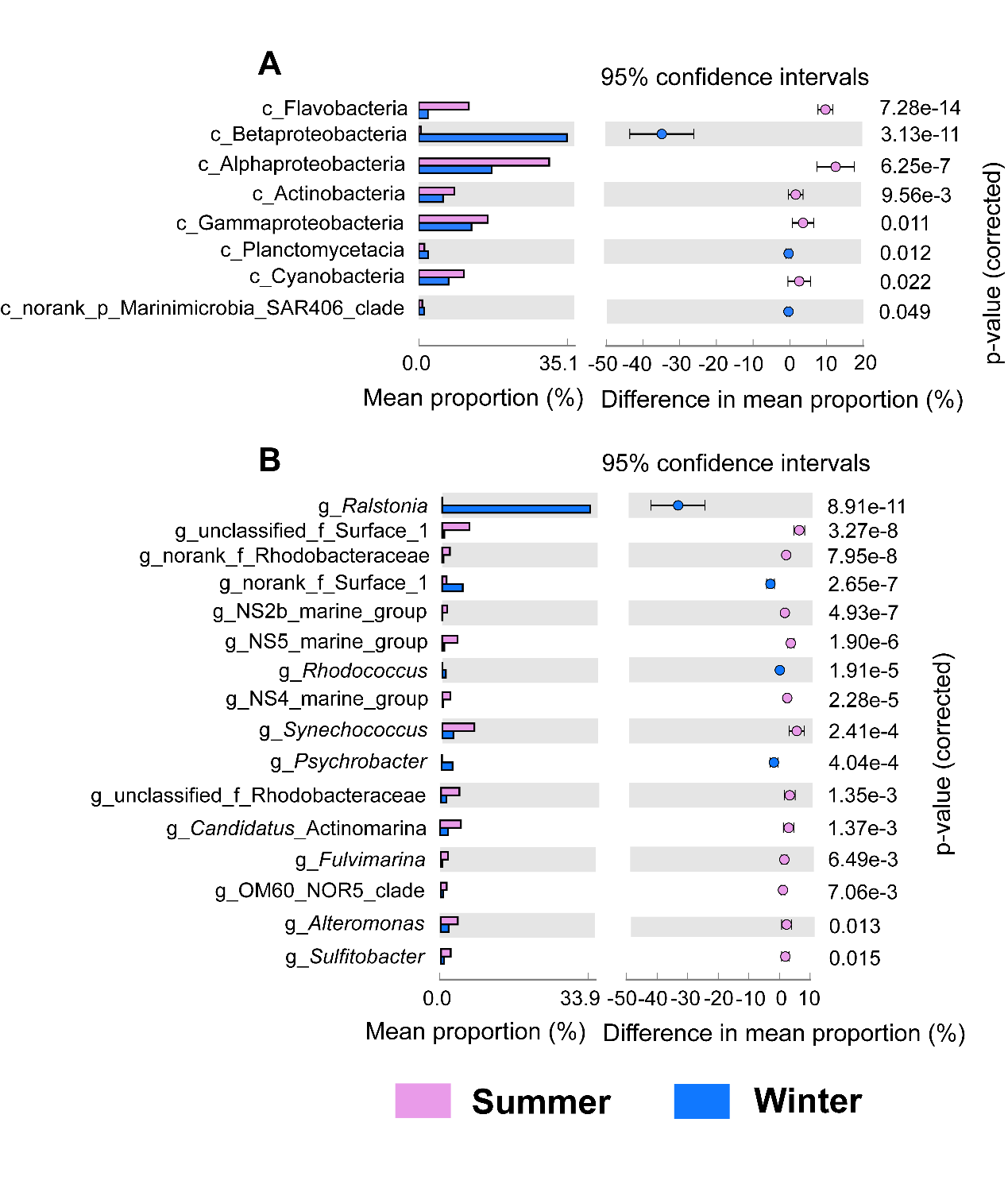


**Fig.S2** Differences in microbial community composition between summer and winter. **(A)**, at class level; **(B)**, at genus level.


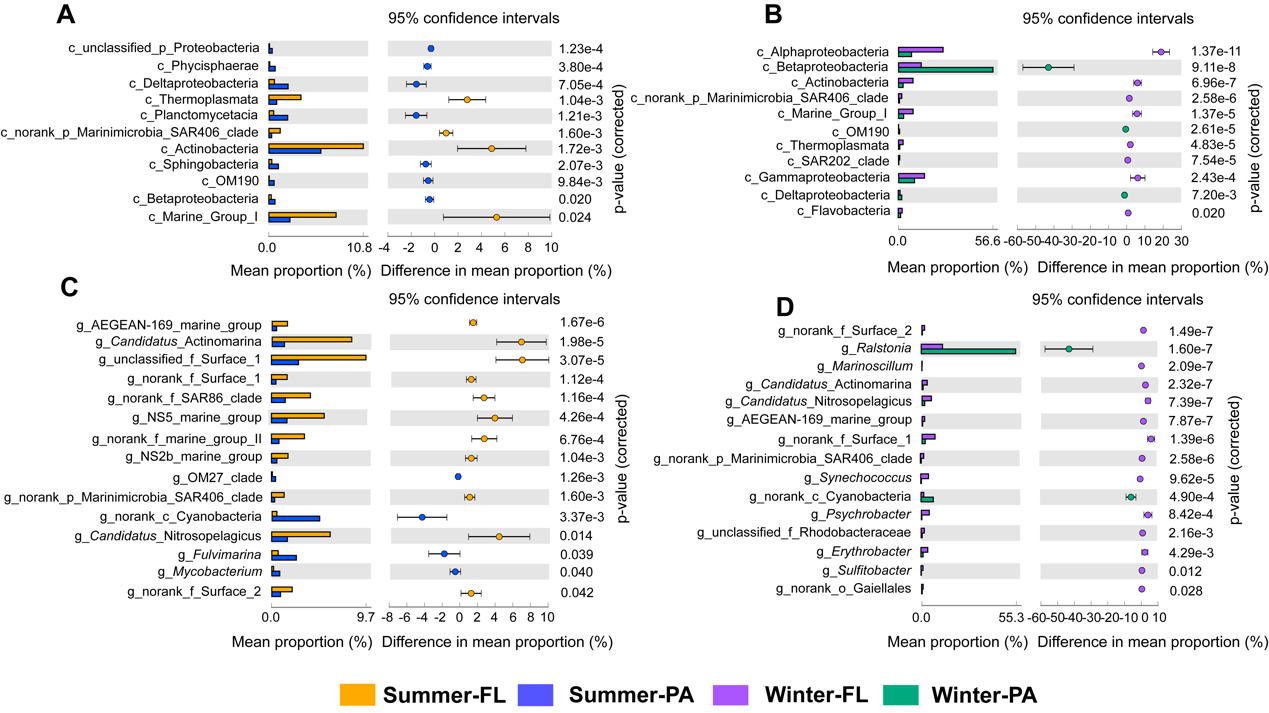


**Fig.S3** Differences in community composition between lifestyles. FL: Free-living; PA: Particle-associated. **(A)**, FL and PA communities at class level in summer; **(B)**, FL and PA communities at class level in winter; **(C)**, FL and PA communities at genus level in summer; **(D)**, FL and PA communities at genus level in winter.


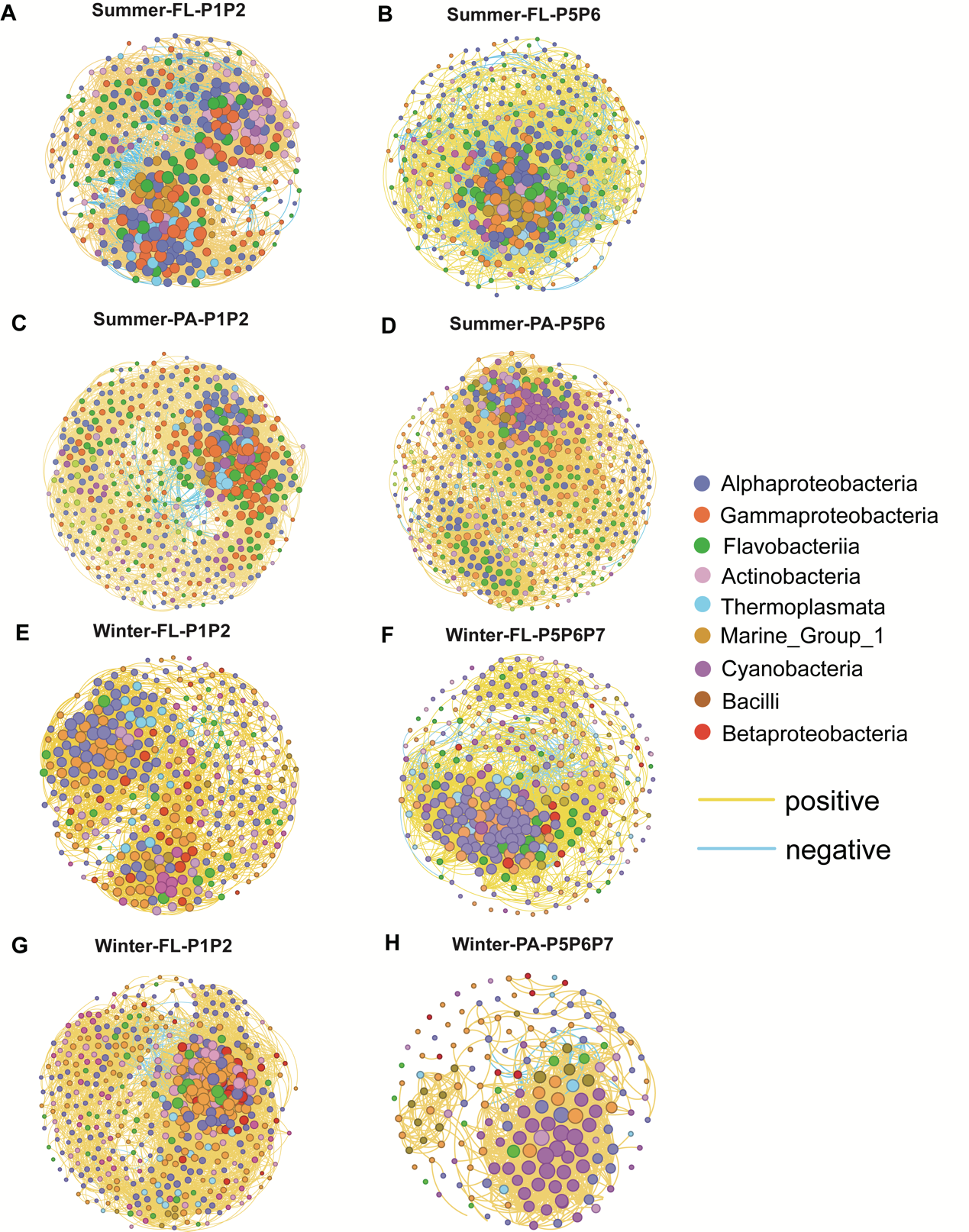


**Fig.S4** Co-occurrence networks of the microbial community in different season and lifestyle group. Each connection shown has a correlation coefficient > |.7| and a *p* value <.01. The size of each node is proportional to the number of connections. The OTUs were colored by subcommunity and taxonomy, respectively. FL: Free-living; PA: Particle-associated.

Supplementary tables

**Table S1** The environmental parameters and microplankton of summer samples from the PN section.

| Station | Longitude  (°E) | Latitude  (°N) | Depth  (m) | Salinity  (PSU) | Temp  (℃) | pH | DO  (mg/l) | Chl *a*  (μg/l) | SiO_3_^2-^  (μM) | NO_2_^-^  (μM) | NH_4_^+^  (μM) | PO_4_^3-^  (μM) | NO_3_^-^  (μM) | DOC  (µM) | SYN  (cells/mL) | PEUK  (cells/mL) | HB  (cells/mL) |
| --- | --- | --- | --- | --- | --- | --- | --- | --- | --- | --- | --- | --- | --- | --- | --- | --- | --- |
| P1_0m | 122.61 | 31.33 | 3.00 | 26.51 | 23.28 | 8.12 | 6.56 | 7.73 | 26.30 | 0.81 | 0.40 | 0.18 | 22.90 | 80.52 | 3962 | 286 | 1155648 |
| P1_10m | 122.61 | 31.33 | 9.60 | 30.97 | 20.80 | 7.97 | 3.89 | 5.99 | 22.00 | 0.83 | 0.08 | 0.44 | 17.30 | 74.51 | 2932 | 316 | 467856 |
| P1_30m | 122.61 | 31.33 | 33.60 | 34.16 | 20.24 | 7.98 | 3.64 | 0.84 | 18.90 | 0.14 | N.D. | 0.68 | 11.40 | 52.32 | 330 | 12 | 284976 |
| P2_0m | 123.13 | 30.92 | 2.60 | 31.74 | 27.02 | 8.29 | 7.84 | 2.47 | 5.49 | 0.03 | 0.18 | 0.05 | 0.25 | 87.33 | 48048 | 6 | 1862496 |
| P2_10m | 123.13 | 30.92 | 11.00 | 32.61 | 25.76 | 8.25 | 6.47 | 0.83 | 5.30 | 0.04 | 0.22 | 0.05 | 0.80 | 119.5 | 39692 | 0 | 1103520 |
| P2_30m | 123.13 | 30.92 | 30.70 | 34.31 | 19.27 | 8.00 | 5.57 | 0.35 | 19.70 | 0.30 | 0.02 | 0.78 | 12.30 | 59.31 | 596 | 6 | 260064 |
| P2_50m | 123.13 | 30.92 | 48.60 | 34.32 | 19.25 | 8.00 | 5.48 | 1.32 | 19.90 | 0.28 | 0.03 | 0.75 | 12.30 | 43.64 | 22436 | 2 | 2494464 |
| P3_0m | 123.60 | 30.40 | 3.30 | 31.74 | 27.76 | 8.29 | 6.95 | 1.35 | 3.22 | 0.04 | 0.08 | 0.05 | N.D. | 103.08 | 8104 | 10 | 313776 |
| P3_10m | 123.60 | 30.40 | 12.10 | 32.13 | 27.51 | 8.29 | 6.67 | 0.95 | 3.10 | 0.03 | 0.19 | 0.05 | 0.10 | 101.00 | 5424 | 4 | 440208 |
| P3_30m | 123.60 | 30.40 | 30.90 | 34.33 | 23.80 | 8.10 | 5.26 | 0.56 | 5.00 | 0.16 | 0.05 | 0.06 | 1.50 | 88.08 | 4950 | 8 | 140544 |
| P3_60m | 123.60 | 30.40 | 60.20 | 34.45 | 20.50 | 8.04 | 4.4 | 0.27 | 14.80 | 0.15 | 0.01 | 0.52 | 8.60 | 68.60 | 584 | 4 | 188112 |
| P4_0m | 124.30 | 30.00 | 5.00 | 33.08 | 28.10 | 8.31 | 7.91 | 1.21 | 1.14 | 0.05 | 0.07 | 0.04 | N.D. | 76.70 | 10036 | 6 | 383376 |
| P4_10m | 124.30 | 30.00 | 11.70 | 33.08 | 28.10 | 8.31 | 7.96 | 1.75 | 1.00 | 0.01 | 0.04 | 0.04 | N.D. | 80.53 | 11866 | 30 | 366816 |
| P4_30m | 124.30 | 30.00 | 31.30 | 33.57 | 26.70 | 8.17 | 8.25 | 0.67 | 1.00 | 0.02 | 0.02 | 0.04 | N.D. | 101.92 | 3020 | 10 | 242400 |
| P4_50m | 124.30 | 30.00 | 51.10 | 34.31 | 23.57 | 8.11 | 5.53 | 1.08 | 6.90 | 0.10 | 0.01 | 0.23 | 2.00 | 73.22 | 2134 | 8 | 137520 |
| P5_0m | 124.90 | 29.60 | 4.20 | 33.85 | 26.92 | 8.20 | 6.81 | 0.28 | 0.80 | N.D. | N.D. | 0.02 | N.D. | 81.50 | 7139 | 698 | 778384 |
| P5_10m | 124.90 | 29.60 | 11.40 | 33.84 | 26.90 | 8.20 | 6.85 | 0.26 | 0.80 | N.D. | 0.04 | 0.01 | N.D. | 81.66 | 4067 | 43 | 2833959 |
| P5_30m | 124.90 | 29.60 | 31.90 | 34.01 | 23.72 | 8.21 | 6.88 | 1.64 | 3.50 | N.D. | N.D. | 0.02 | N.D. | 59.24 | 31439 | 94 | 2256539 |
| P5_50m | 124.90 | 29.60 | 51.20 | 34.22 | 20.24 | 8.08 | 4.85 | 0.95 | 12.00 | 0.06 | N.D. | 0.48 | 7.40 | 63.67 | 8202 | 90 | 1197208 |
| P5_80m | 124.90 | 29.60 | 79.80 | 34.26 | 19.86 | 8.08 | 4.65 | 0.24 | 13.60 | 0.00 | N.D. | 0.56 | 8.60 | 71.63 | 1014 | 51 | 450955 |
| P6_0m | 125.50 | 29.12 | 2.40 | 33.58 | 27.45 | 8.18 | 6.79 | 0.19 | 0.90 | N.D. | 0.01 | 0.02 | N.D. | 81.39 | 2106 | 84 | 477894 |
| P6_10m | 125.50 | 29.12 | 11.70 | 33.59 | 27.47 | 8.19 | 6.65 | 0.17 | 0.90 | N.D. | 0.01 | 0.00 | N.D. | 74.08 | 1929 | 16 | 692473 |
| P6_30m | 125.50 | 29.12 | 31.80 | 33.99 | 25.39 | 8.19 | 7.18 | 0.24 | 0.90 | N.D. | N.D. | 0.02 | N.D. | 64.64 | 3031 | 510 | 316800 |
| P6_50m | 125.50 | 29.12 | 51.30 | 34.16 | 20.78 | 8.22 | 7.51 | 0.83 | 12.00 | N.D. | N.D. | 0.03 | 0.00 | 81.68 | 2792 | 1245 | 730286 |
| P6_75m | 125.50 | 29.12 | 72.80 | 34.51 | 18.58 | 8.07 | 4.66 | 0.45 | 17.10 | 0.00 | N.D. | 0.75 | 11.2 | 62.26 | 465 | 12 | 626743 |
| P6_90m | 125.50 | 29.12 | 89.80 | 34.54 | 18.45 | 8.06 | 4.66 | 0.33 | 18.20 | 0.02 | N.D. | 0.77 | 11.3 | 60.88 | 380 | 39 | 377731 |

*^a^* N.D. represents not done.

*^b^* Chl *a* data were provided by Yahui Gao of Xiamen University; nutrient measurements were provided by Yu Xin of Ocean University of China; pH data were provided by Chunying Liu of Ocean University of China; DO and DOC data were provided by Guipeng Yang of Ocean University of China.

**Table S2** The environmental parameters and microplankton of winter samples from the PN section.

| Station | Longitude  (°E) | Latitude  (°N) | Depth  (m) | Salinity  (PSU) | Temp  (℃) | pH | DO  (mg/l) | Chl *a*  (μg/l) | SiO_3_^2-^  (μM) | NO_2_^-^  (μM) | PO_4_^3-^  (μM) | NO_3_^-^  (μM) | DOC  (µM) | SYN  (cells/mL) | PRO  (cells/mL) | PEUK  (cells/mL) | HB  (cells/mL) |
| --- | --- | --- | --- | --- | --- | --- | --- | --- | --- | --- | --- | --- | --- | --- | --- | --- | --- |
| P1_0m | 122.72 | 30.96 | 3.00 | 30.61 | 13.96 | 8.17 | 8.46 | 0.20 | 22.96 | 0.11 | 0.36 | 17.20 | 121.17 | 400 | 298 | 175 | 370000 |
| P1_10m | 122.72 | 30.96 | 10.00 | 30.88 | 14.21 | 8.15 | 8.29 | 0.17 | 21.67 | 0.06 | 0.61 | 15.89 | 92.50 | 164 | 171 | 58 | 492000 |
| P1_20m | 122.72 | 30.96 | 19.00 | 31.88 | 14.80 | 8.15 | 8.25 | 0.14 | 21.29 | 0.11 | 0.55 | 15.74 | 113.83 | 18 | 0 | 4 | 314000 |
| P2_0m | 123.01 | 30.87 | 2.00 | 33.04 | 15.76 | 8.17 | 7.94 | 0.46 | 14.54 | 0.07 | 0.52 | 9.04 | 109.42 | 720 | 196 | 218 | 480000 |
| P2_10m | 123.01 | 30.87 | 11.00 | 33.04 | 15.76 | 8.15 | 7.93 | 0.54 | 13.92 | 0.06 | 0.50 | 8.73 | 136.92 | 342 | 124 | 22 | 1450000 |
| P2_30m | 123.01 | 30.87 | 30.00 | 33.15 | 16.06 | 8.15 | 8.14 | 0.39 | 14.06 | 0.06 | 0.52 | 8.57 | 88.58 | 476 | 178 | 113 | 370000 |
| P2_50m | 123.01 | 30.87 | 47.00 | 33.16 | 16.10 | 8.15 | 7.94 | 0.35 | 14.03 | 0.06 | 0.53 | 8.62 | 81.38 | 585 | 156 | 124 | 195000 |
| P3_0m | 123.75 | 30.37 | 3.00 | 33.96 | 17.48 | 8.14 | 7.86 | 0.31 | 11.06 | 0.09 | 0.45 | 5.55 | 99.62 | 1022 | 156 | 87 | 347000 |
| P3_10m | 123.75 | 30.37 | 10.00 | 33.96 | 17.49 | 8.14 | 7.72 | 0.40 | 10.71 | 0.09 | 0.44 | 5.28 | 76.51 | 1196 | 95 | 98 | 194000 |
| P3_30m | 123.75 | 30.37 | 31.00 | 33.97 | 17.50 | 8.13 | 7.72 | 0.25 | 10.96 | 0.09 | 0.44 | 5.39 | 71.64 | 76 | 4 | 4 | 328000 |
| P3_50m | 123.75 | 30.37 | 53.00 | 33.97 | 17.49 | 8.14 | 7.77 | 0.42 | 10.95 | 0.09 | 0.45 | 5.40 | 72.20 | 36 | 4 | 4 | 425000 |
| P4_0m | 124.55 | 29.85 | 4.00 | 33.88 | 17.61 | 8.12 | 7.56 | 0.32 | 9.94 | 0.09 | 0.43 | 5.19 | 87.42 | 989 | 80 | 385 | 527000 |
| P4_10m | 124.55 | 29.85 | 10.00 | 33.88 | 17.61 | 8.12 | 7.27 | 0.39 | 9.95 | 0.10 | 0.43 | 5.28 | 91.17 | 1455 | 160 | 269 | 304000 |
| P4_30m | 124.55 | 29.85 | 30.00 | 33.88 | 17.61 | 8.12 | 7.30 | 0.34 | 9.88 | 0.10 | 0.43 | 5.23 | 74.48 | 2364 | 44 | 1167 | 326000 |
| P4_50m | 124.55 | 29.85 | 50.00 | 33.88 | 17.61 | 8.12 | 7.17 | 0.32 | 10.04 | 0.11 | 0.44 | 5.39 | 62.90 | 2247 | 84 | 1138 | 206000 |
| P4_60m | 124.55 | 29.85 | 62.00 | 33.88 | 17.61 | 8.12 | 7.02 | 0.40 | 9.94 | 0.10 | 0.43 | 5.31 | 75.13 | 2040 | 76 | 880 | 196000 |
| P5_0m | 125.39 | 29.27 | 4.00 | 34.19 | 18.97 | 8.14 | 7.39 | 0.33 | 7.98 | 0.03 | 0.33 | 3.98 | 84.08 | 2825 | 145 | 516 | 250000 |
| P5_10m | 125.39 | 29.27 | 10.00 | 34.19 | 18.98 | 8.14 | 7.36 | 0.34 | 8.12 | 0.06 | 0.33 | 4.15 | 93.56 | 1513 | 45 | 113 | 267000 |
| P5_30m | 125.39 | 29.27 | 30.00 | 34.19 | 18.98 | 8.14 | 7.28 | 0.33 | 8.20 | 0.01 | 0.31 | 3.70 | 89.83 | 1321 | 60 | 60 | 285000 |
| P5_50m | 125.39 | 29.27 | 50.00 | 34.19 | 18.99 | 8.13 | 7.25 | 0.33 | 8.27 | 0.04 | 0.34 | 4.22 | 74.44 | 543 | 4 | 102 | 299000 |
| P5_90m | 125.39 | 29.27 | 87.00 | 34.19 | 18.99 | 8.13 | 7.28 | 0.17 | 8.26 | 0.06 | 0.33 | 4.32 | 75.32 | 1242 | 11 | 11 | 407000 |
| P6_0m | 126.15 | 28.70 | 4.00 | 34.51 | 20.62 | 8.19 | 7.13 | 0.51 | 3.25 | 0.40 | 0.12 | 0.89 | 81.67 | 5196 | 219 | 355 | 317000 |
| P6_10m | 126.15 | 28.70 | 10.00 | 34.51 | 20.62 | 8.17 | 7.19 | 0.50 | 3.25 | 0.33 | 0.09 | 0.66 | 68.64 | 4853 | 189 | 423 | 299000 |
| P6_30m | 126.15 | 28.70 | 30.00 | 34.50 | 20.55 | 8.17 | 7.03 | 0.42 | 3.34 | 0.46 | 0.14 | 0.98 | 70.22 | 4230 | 449 | 162 | 282000 |
| P6_50m | 126.15 | 28.70 | 50.00 | 34.50 | 20.53 | 8.16 | 7.09 | 0.30 | 3.44 | 0.33 | 0.11 | 0.89 | 74.39 | 3174 | 238 | 415 | 311000 |
| P6_75m | 126.15 | 28.70 | 75.00 | 34.50 | 20.51 | 8.16 | 7.20 | 0.26 | 3.56 | 0.50 | 0.13 | 1.12 | 74.53 | 3664 | 181 | 785 | 300000 |
| P6_120m | 126.15 | 28.70 | 121.00 | 34.50 | 20.48 | 8.16 | 7.03 | 0.30 | 3.66 | 0.47 | 0.14 | 1.14 | 53.13 | 2494 | 60 | 94 | 354000 |
| P7_0m | 127.00 | 28.15 | 3.00 | 34.66 | 23.31 | 8.18 | 7.00 | 0.23 | 1.32 | 0.01 | 0.12 | 0.89 | 69.54 | 389 | 223 | 94 | 195000 |
| P7_50m | 127.00 | 28.15 | 50.00 | 34.66 | 23.33 | 8.18 | 7.05 | 0.24 | 1.38 | 0.01 | 0.01 | 0.02 | 81.79 | 9385 | 6226 | 2200 | 161000 |
| P7_100m | 127.00 | 28.15 | 101.00 | 34.71 | 23.21 | 8.17 | 6.99 | 0.23 | 1.42 | 0.01 | 0.12 | 6.89 | 51.94 | 1117 | 928 | 113 | 201000 |
| P7_300m | 127.00 | 28.15 | 298.00 | 34.64 | 15.95 | 8.08 | 5.96 | 0.00 | 14.26 | 0.01 | 0.76 | 11.13 | 60.67 | 23 | 34 | 11 | 51400 |
| P7_500m | 127.00 | 28.15 | 498.00 | 34.28 | 8.66 | 7.88 | 4.25 | 0.00 | 56.61 | 0.01 | 2.13 | 29.77 | 42.23 | 11 | 8 | 8 | 26500 |
| P7_750m | 127.00 | 28.15 | 750.00 | 34.35 | 5.71 | 7.81 | 3.08 | 0.01 | 92.33 | 0.01 | 2.72 | 37.20 | 44.76 | 8 | 4 | 4 | 31500 |
| P7_900m | 127.00 | 28.15 | 899.00 | 34.37 | 5.07 | 7.79 | 2.76 | 0.00 | 101.49 | 0.01 | 2.82 | 38.42 | 45.81 | 60 | 11 | 0 | 34000 |

*^a^* N.D. represents not done.

*^b^* Chl a data were provided by Yahui Gao of Xiamen University; nutrient measurements were provided by Yu Xin of Ocean University of China; pH data were provided by Chunying Liu of Ocean University of China; DO and DOC data were provided by Guipeng Yang of Ocean University of China.

**Table S3** The original sequence numbers, the OTU numbers after subsampling and the alpha diversity of summer samples along the PN section.

| Samples | Sequence numbers | OTU | Shannon | Chao 1 | coverage |
| --- | --- | --- | --- | --- | --- |
| P1_0m_02 | 53067 | 810 | 4.25 | 1067.11 | 99.05% |
| P1_10m_02 | 43251 | 806 | 4.24 | 1231.13 | 98.89% |
| P1_30m_02 | 31223 | 1127 | 4.72 | 1737.84 | 98.38% |
| P2_0m_02 | 33700 | 391 | 3.48 | 629.00 | 99.48% |
| P2_10m_02 | 74416 | 413 | 3.53 | 585.02 | 99.54% |
| P2_30m_02 | 33398 | 914 | 4.53 | 1329.56 | 98.82% |
| P2_50m_02 | 60215 | 565 | 3.80 | 915.50 | 99.19% |
| P3_0m_02 | 61892 | 737 | 4.42 | 1111.35 | 99.04% |
| P3_10m_02 | 64488 | 612 | 4.44 | 831.48 | 99.35% |
| P3_30m_02 | 30264 | 783 | 4.72 | 1256.17 | 99.00% |
| P3_60m_02 | 73812 | 1177 | 4.82 | 1635.45 | 98.47% |
| P4_0m_02 | 36306 | 943 | 4.44 | 1115.98 | 99.21% |
| P4_10m_02 | 61365 | 537 | 4.16 | 734.15 | 99.42% |
| P4_30m_02 | 47044 | 600 | 3.63 | 886.07 | 99.25% |
| P4_50m_02 | 37476 | 936 | 4.69 | 1422.63 | 98.80% |
| P5_0m_02 | 34510 | 617 | 4.16 | 877.89 | 99.29% |
| P5_10m_02 | 70602 | 591 | 4.13 | 793.67 | 99.39% |
| P5_30m_02 | 32955 | 486 | 3.58 | 706.37 | 99.42% |
| P5_50m_02 | 61382 | 734 | 3.82 | 1120.23 | 98.92% |
| P5_80m_02 | 91706 | 997 | 4.51 | 1427.52 | 98.74% |
| P6_0m_02 | 69666 | 706 | 4.31 | 1013.71 | 99.14% |
| P6_10m_02 | 80497 | 605 | 3.83 | 874.15 | 99.26% |
| P6_30m_02 | 69690 | 811 | 4.43 | 1273.00 | 98.90% |
| P6_50m_02 | 50854 | 813 | 4.19 | 1255.69 | 98.90% |
| P6_75m_02 | 74328 | 886 | 4.20 | 1315.22 | 98.82% |
| P6_90m_02 | 46077 | 931 | 4.42 | 1420.16 | 98.73% |
| P1_0m_3 | 64489 | 857 | 4.17 | 1034.89 | 99.14% |
| P1_10m_3 | 32297 | 1202 | 4.88 | 1502.14 | 98.72% |
| P1_30m_3 | 61387 | 1763 | 4.95 | 2816.33 | 97.11% |
| P2_0m_3 | 38740 | 813 | 4.92 | 939.67 | 99.42% |
| P2_10m_3 | 73701 | 910 | 4.46 | 1037.57 | 99.39% |
| P2_30m_3 | 50540 | 1703 | 5.39 | 2647.28 | 97.50% |
| P2_50m_3 | 45562 | 1478 | 4.80 | 2159.95 | 97.87% |
| P3_0m_3 | 33241 | 720 | 3.94 | 809.69 | 99.44% |
| P3_10m_3 | 52132 | 878 | 4.90 | 968.60 | 99.52% |
| P3_30m_3 | 32270 | 912 | 4.99 | 1155.16 | 99.09% |
| P3_60m_3 | 60839 | 2028 | 5.30 | 3318.43 | 96.66% |
| P4_0m_3 | 32742 | 490 | 3.84 | 702.30 | 99.42% |
| P4_10m_3 | 74684 | 982 | 4.85 | 1240.07 | 99.02% |
| P4_30m_3 | 32494 | 1063 | 4.71 | 1277.11 | 99.01% |
| P4_50m_3 | 72754 | 2203 | 5.50 | 3159.72 | 96.82% |
| P5_0m_3 | 31824 | 520 | 3.15 | 586.90 | 99.64% |
| P5_10m_3 | 70116 | 1253 | 5.42 | 1439.50 | 99.04% |
| P5_30m_3 | 45749 | 1168 | 4.56 | 1409.55 | 98.86% |
| P5_50m_3 | 32065 | 1283 | 5.06 | 1601.64 | 98.67% |
| P5_80m_3 | 36751 | 1166 | 5.47 | 1227.17 | 99.55% |
| P6_0m_3 | 35203 | 744 | 4.34 | 1017.64 | 99.15% |
| P6_10m_3 | 30459 | 618 | 3.88 | 880.69 | 99.23% |
| P6_30m_3 | 74376 | 1776 | 5.32 | 2481.34 | 97.57% |
| P6_50m_3 | 33927 | 1031 | 5.12 | 1173.62 | 99.27% |
| P6_75m_3 | 71282 | 1356 | 4.66 | 1755.12 | 98.44% |
| P6_90m_3 | 40221 | 2072 | 5.80 | 2751.42 | 97.42% |

*^a^* “02” of the sample name means the free-living samples, and “3” means the particle-associated samples.

**Table S4** The original sequence numbers, the OTU numbers after subsampling and the alpha diversity of winter samples along the PN section.

| Samples | Sequence numbers | OTU | Shannon | Chao 1 | coverage |
| --- | --- | --- | --- | --- | --- |
| P1_0m_02 | 63055 | 2050 | 5.11 | 2773.60 | 97.27% |
| P1_10m_02 | 73416 | 1696 | 4.46 | 2554.60 | 97.26% |
| P1_20m_02 | 73021 | 1583 | 4.70 | 2617.69 | 97.42% |
| P2_0m_02 | 74476 | 927 | 3.83 | 1786.67 | 98.36% |
| P2_10m_02 | 72899 | 993 | 4.18 | 1880.14 | 98.33% |
| P2_30m_02 | 56074 | 1078 | 3.73 | 1918.45 | 98.19% |
| P2_50m_02 | 74959 | 1070 | 4.37 | 1940.78 | 98.23% |
| P3_0m_02 | 54187 | 433 | 4.02 | 492.91 | 99.81% |
| P3_10m_02 | 31643 | 923 | 4.95 | 1134.92 | 99.10% |
| P3_30m_02 | 67205 | 763 | 4.14 | 1138.79 | 98.97% |
| P3_50m_02 | 73488 | 849 | 4.53 | 1275.88 | 98.93% |
| P4_0m_02 | 71264 | 1052 | 4.89 | 1333.49 | 98.80% |
| P4_10m_02 | 71430 | 1190 | 4.94 | 1733.06 | 98.38% |
| P4_30m_02 | 71973 | 921 | 4.97 | 1251.83 | 98.90% |
| P4_50m_02 | 71819 | 1948 | 5.82 | 2598.27 | 97.58% |
| P4_60m_02 | 69910 | 785 | 4.81 | 905.48 | 99.37% |
| P5_0m_02 | 72373 | 1912 | 5.61 | 2683.58 | 97.45% |
| P5_10m_02 | 62566 | 878 | 4.61 | 1213.62 | 98.93% |
| P5_30m_02 | 70438 | 738 | 4.17 | 979.09 | 99.15% |
| P5_50m_02 | 68699 | 765 | 4.86 | 858.93 | 99.51% |
| P5_90m_02 | 68154 | 614 | 4.29 | 685.23 | 99.65% |
| P6_0m_02 | 69316 | 776 | 4.80 | 1053.58 | 99.15% |
| P6_10m_02 | 70051 | 804 | 4.62 | 1109.43 | 99.07% |
| P6_30m_02 | 73321 | 753 | 4.35 | 1151.18 | 99.05% |
| P6_50m_02 | 73579 | 1180 | 4.79 | 1841.14 | 98.16% |
| P6_75m_02 | 69382 | 728 | 3.73 | 1099.93 | 98.93% |
| P6_120m_02 | 61716 | 552 | 4.61 | 582.00 | 99.82% |
| P7_0m_02 | 45404 | 523 | 3.66 | 583.86 | 99.71% |
| P7_50m_02 | 45552 | 424 | 4.10 | 453.00 | 99.87% |
| P7_100m_02 | 47754 | 492 | 2.64 | 551.51 | 99.60% |
| P7_300m_02 | 57755 | 1069 | 3.85 | 1361.46 | 98.87% |
| P7_500m_02 | 69786 | 644 | 2.42 | 947.56 | 99.12% |
| P7_750m_02 | 61626 | 679 | 3.34 | 805.26 | 99.37% |
| P7_120m_02 | 59442 | 395 | 0.78 | 582.07 | 99.34% |
| P1_0m_3 | 72209 | 2530 | 5.74 | 3707.84 | 96.14% |
| P1_10m_3 | 71764 | 2558 | 6.01 | 4059.69 | 95.95% |
| P1_20m_3 | 73607 | 2607 | 5.81 | 4065.18 | 95.85% |
| P2_0m_3 | 72806 | 1076 | 4.67 | 1203.89 | 99.23% |
| P2_10m_3 | 38655 | 1543 | 4.95 | 2011.38 | 98.12% |
| P2_30m_3 | 72251 | 2077 | 5.29 | 3502.76 | 96.43% |
| P2_50m_3 | 69552 | 2386 | 5.75 | 3839.25 | 96.12% |
| P3_0m_3 | 66531 | 1317 | 3.30 | 1923.75 | 98.01% |
| P3_10m_3 | 74147 | 1326 | 4.08 | 2052.20 | 98.00% |
| P3_30m_3 | 72618 | 330 | 0.66 | 432.29 | 99.56% |
| P3_50m_3 | 71520 | 835 | 1.81 | 1068.01 | 98.94% |
| P4_0m_3 | 72882 | 1047 | 3.41 | 1452.51 | 98.53% |
| P4_10m_3 | 69315 | 1754 | 5.21 | 2329.43 | 97.74% |
| P4_30m_3 | 66428 | 1003 | 2.46 | 1529.64 | 98.38% |
| P4_50m_3 | 70878 | 1158 | 2.63 | 1640.17 | 98.31% |
| P4_60m_3 | 74120 | 968 | 2.52 | 1477.41 | 98.39% |
| P5_0m_3 | 73681 | 353 | 1.14 | 433.73 | 99.66% |
| P5_10m_3 | 69033 | 959 | 2.79 | 1415.79 | 98.53% |
| P5_30m_3 | 58921 | 645 | 1.71 | 960.86 | 99.02% |
| P5_50m_3 | 71091 | 620 | 1.56 | 908.31 | 99.09% |
| P5_60m_3 | 55709 | 270 | 0.56 | 316.75 | 99.72% |
| P6_0m_3 | 72094 | 628 | 1.49 | 822.01 | 99.22% |
| P6_10m_3 | 74560 | 439 | 1.00 | 637.00 | 99.38% |
| P6_30m_3 | 73204 | 544 | 1.29 | 728.42 | 99.28% |
| P6_50m_3 | 73757 | 542 | 1.16 | 789.31 | 99.19% |
| P6_75m_3 | 45060 | 378 | 0.81 | 469.04 | 99.54% |
| P6_120m_3 | 74179 | 1295 | 3.83 | 1805.11 | 98.27% |
| P7_0m_3 | 61752 | 428 | 2.17 | 473.22 | 99.76% |
| P7_50m_3 | 59677 | 278 | 0.78 | 276.46 | 99.89% |
| P7_100m_3 | 49493 | 200 | 0.43 | 301.23 | 99.67% |
| P7_300m_3 | 71552 | 648 | 1.77 | 673.66 | 99.63% |
| P7_500m_3 | 50429 | 74 | 0.23 | 104.08 | 99.91% |
| P7_750m_3 | 55763 | 309 | 0.64 | 445.00 | 99.52% |
| P7_900m_3 | 50308 | 588 | 2.23 | 838.53 | 99.19% |

*^a^* “02” of the sample name means the free-living samples, and “3” means the particle-associated samples.

**Table S5** Detailed results of the mantel test between different environmental factors and microbial communities.

| group | environment factors | r | p.value | rd | pd |
| --- | --- | --- | --- | --- | --- |
| SummerFL | Longtitude | 0.1139313 | 0.050 | < 0.2 | 0.01 - 0.05 |
|  | Latitude | 0.10863994 | 0.071 | < 0.2 | >= 0.05 |
|  | Depth | 0.42330671 | 0.001 | >= 0.4 | < 0.01 |
|  | Salinity | 0.31930326 | 0.003 | 0.2 - 0.4 | < 0.01 |
|  | Temp | 0.49114671 | 0.001 | >= 0.4 | < 0.01 |
|  | pH | 0.32011948 | 0.001 | 0.2 - 0.4 | < 0.01 |
|  | DO | 0.30057295 | 0.001 | 0.2 - 0.4 | < 0.01 |
|  | Chl-*a* | 0.15829866 | 0.113 | < 0.2 | >= 0.05 |
|  | SiO_3_^2-^ | 0.35972012 | 0.001 | 0.2 - 0.4 | < 0.01 |
|  | PO_4_^3-^ | 0.24851992 | 0.006 | 0.2 - 0.4 | < 0.01 |
|  | SYN | 0.07578251 | 0.213 | < 0.2 | >= 0.05 |
|  | PRO | -0.1218513 | 0.898 | < 0.2 | >= 0.05 |
|  | PEUL | -0.0284859 | 0.561 | < 0.2 | >= 0.05 |
| SummerPA | Longtitude | 0.09905823 | 0.077 | < 0.2 | >= 0.05 |
|  | Latitude | 0.08611528 | 0.119 | < 0.2 | >= 0.05 |
|  | Depth | 0.08511787 | 0.154 | < 0.2 | >= 0.05 |
|  | Salinity | 0.08572358 | 0.175 | < 0.2 | >= 0.05 |
|  | Temp | 0.17670658 | 0.003 | < 0.2 | < 0.01 |
|  | pH | 0.18946622 | 0.009 | < 0.2 | < 0.01 |
|  | DO | 0.1541557 | 0.037 | < 0.2 | 0.01 - 0.05 |
|  | Chl-*a* | 0.03592704 | 0.277 | < 0.2 | >= 0.05 |
|  | SiO_3_^2-^ | 0.14106849 | 0.056 | < 0.2 | >= 0.05 |
|  | PO_4_^3-^ | 0.13156865 | 0.084 | < 0.2 | >= 0.05 |
|  | SYN | -0.0186835 | 0.484 | < 0.2 | >= 0.05 |
|  | PRO | -0.0536263 | 0.612 | < 0.2 | >= 0.05 |
|  | PEUL | 0.15556329 | 0.092 | < 0.2 | >= 0.05 |
| WinterFL | Longitude | 0.33340326 | 0.001 | 0.2 - 0.4 | < 0.01 |
|  | Latitude | 0.33592424 | 0.001 | 0.2 - 0.4 | < 0.01 |
|  | Depth | 0.46466773 | 0.001 | >= 0.4 | < 0.01 |
|  | Salinity | 0.05587918 | 0.265 | < 0.2 | >= 0.05 |
|  | Temp | 0.46164934 | 0.001 | >= 0.4 | < 0.01 |
|  | pH | 0.403199 | 0.001 | >= 0.4 | < 0.01 |
|  | DO | 0.44670069 | 0.001 | >= 0.4 | < 0.01 |
|  | Chl-*a* | 0.33607785 | 0.001 | 0.2 - 0.4 | < 0.01 |
|  | SiO_3_^2-^ | 0.4223997 | 0.001 | >= 0.4 | < 0.01 |
|  | NO_2_^-^ | 0.06354451 | 0.221 | < 0.2 | >= 0.05 |
|  | PO_4_^3-^ | 0.42450201 | 0.001 | >= 0.4 | < 0.01 |
|  | NO_3_^-^ | 0.4211503 | 0.001 | >= 0.4 | < 0.01 |
|  | DOC | 0.21901469 | 0.016 | 0.2 - 0.4 | 0.01 - 0.05 |
|  | SYN | 0.0685429 | 0.217 | < 0.2 | >= 0.05 |
|  | PRO | 0.09614473 | 0.174 | < 0.2 | >= 0.05 |
|  | PEUL | 0.06789932 | 0.246 | < 0.2 | >= 0.05 |
|  | HB | 0.08080032 | 0.222 | < 0.2 | >= 0.05 |
| WinterPA | Longitude | 0.57335997 | 0.001 | >= 0.4 | < 0.01 |
|  | Latitude | 0.55088042 | 0.001 | >= 0.4 | < 0.01 |
|  | Depth | -0.0834166 | 0.695 | < 0.2 | >= 0.05 |
|  | Salinity | 0.67833678 | 0.001 | >= 0.4 | < 0.01 |
|  | Temp | 0.03239816 | 0.342 | < 0.2 | >= 0.05 |
|  | pH | -0.0961636 | 0.710 | < 0.2 | >= 0.05 |
|  | DO | 0.09571994 | 0.194 | < 0.2 | >= 0.05 |
|  | Chl-*a* | 0.04949159 | 0.269 | < 0.2 | >= 0.05 |
|  | SiO_3_^2-^ | -0.0292951 | 0.509 | < 0.2 | >= 0.05 |
|  | NO_2_^-^ | -0.1500229 | 0.946 | < 0.2 | >= 0.05 |
|  | PO_4_^3-^ | -0.0799459 | 0.684 | < 0.2 | >= 0.05 |
|  | NO_3_^-^ | 0.04944614 | 0.313 | < 0.2 | >= 0.05 |
|  | DOC | 0.44653502 | 0.001 | >= 0.4 | < 0.01 |
|  | SYN | -0.1168479 | 0.878 | < 0.2 | >= 0.05 |
|  | PRO | -0.0905402 | 0.698 | < 0.2 | >= 0.05 |
|  | PEUL | -0.1494934 | 0.949 | < 0.2 | >= 0.05 |
|  | HB | 0.27165864 | 0.013 | 0.2 - 0.4 | 0.01 - 0.05 |

*^a^* FL: Free-living; PA: Particle-associated.

**Table S6** The correlations between bacterial abundances and environmental factors in particle-associated and free-living bacteria in the PN section.

| Environmental  Parameters | | Longitude | Latitude | Salinity | DOC | Chl *a* | pH | DO | PO_4_^3-^ | NH_4_^+^ | NO_3_^-^ |
| --- | --- | --- | --- | --- | --- | --- | --- | --- | --- | --- | --- |
| summer | FL |  |  |  |  | 0.426 |  |  |  |  |  |
|  | PA |  |  |  | **-0.636** |  | -0.460 |  | 0.404 |  | **0.708** |
|  | Total |  |  |  |  | **0.491** |  |  |  |  |  |
| winter | FL |  |  |  |  |  |  |  |  |  |  |
|  | PA | **-0.491** | **0.491** | **-0.437** |  |  |  | **0.457** |  |  |  |
|  | Total |  |  |  |  |  |  |  |  |  | 0.401 |

*^a^* FL: Free-living; PA: Particle-associated.

*^a^* Only significant correlations were shown in table. Red, positive; blue, negative. Bold, *p* < 0.01; regular, *p* < 0.05.

**Table S7** The Spearman correlation test between *Ralstonia* and environmental factors in the PN section.

|  | Summer-FL | Summer-PA | Winter-FL | Winter-PA |
| --- | --- | --- | --- | --- |
| Longtitude | 0.318 | 0.397* | 0.156 | 0.719** |
| Latitude | -0.318 | -0.397* | -0.151 | -0.748** |
| Depth | -0.036 | 0.027 | 0.267 | 0.479** |
| Salinity | -0.102 | -0.209 | 0.042 | 0.774** |
| Temp | 0.436* | 0.144 | -0.423* | 0.471** |
| pH | 0.295 | 0.446* | -0.117 | -0.059 |
| DO | 0.359 | 0.382 | -0.164 | -0.645** |
| Chl *a* | -0.092 | -0.023 | -0.387* | -0.242 |
| SiO_3_^2-^ | -0.440* | -0.288 | 0.421* | -0.446** |
| NO_2_^-^ | -0.277 | -0.628** | -0.582** | -0.194 |
| PO_4_^3-^ | -0.308 | -0.259 | 0.515** | -0.406* |
| NO_3_^-^ | -0.444 | -0.438 | 0.542** | -0.364* |
| DOC | 0.211 | 0.458* | -0.131 | -0.631** |
| SYN | -0.102 | 0.178 | -0.427* | 0.227 |
| PEUK | -0.046 | 0.315 | -0.389* | 0.006 |

*^a^* FL: Free-living; PA: Particle-associated.

*^a^* Only significant correlations were shown in table. Red, positive; blue, negative. **, *p* < 0.01; *, *p* < 0.05.

**Table S8** Keystone OTUs in each co-occurrence network.

| Group | name | degree | betweenness  centrality | taxonomy |
| --- | --- | --- | --- | --- |
| Summer-FL | OTU3584 | 49 | 29.25 | c__Marine_Group_I; o__Unknown_Order_c__Marine_Group_I; f__Unknown_Family_o__Unknown_Order_c__Marine_Group_I; g__Candidatus_Nitrosopelagicus; s__unclassified_g__Candidatus_Nitrosopelagicus |
|  | OTU3064 | 50 | 32.15 | c__Gammaproteobacteria; o__Oceanospirillales; f__SAR86_clade; g__norank_f__SAR86_clade; s__SAR86_cluster_bacterium_REDSEA-S09_B4 |
|  | OTU11027 | 60 | 34.12 | c__Thermoplasmata; o__Thermoplasmatales; f__Marine_Group_II; g__norank_f__Marine_Group_II; s__uncultured_marine_group_II_euryarchaeote |
|  | OTU9275 | 57 | 35.10 | c__Actinobacteria; o__Acidimicrobiales; f__Sva0996_marine_group; g__norank_f__Sva0996_marine_group; s__uncultured_Acidimicrobineae_bacterium_g__norank_f__Sva0996_marine_group |
|  | OTU13152 | 57 | 39.90 | c__Marine_Group_I; o__norank_c__Marine_Group_I; f__norank_c__Marine_Group_I; g__norank_c__Marine_Group_I; s__uncultured_marine_archaeon_g__norank_c__Marine_Group_I |
|  | OTU10905 | 50 | 76.09 | c__Thermoplasmata; o__Thermoplasmatales; f__Marine_Group_II; g__norank_f__Marine_Group_II; s__uncultured_marine_archaeon_g__norank_f__Marine_Group_II |
|  | OTU10903 | 42 | 89.00 | c__Marine_Group_I; o__Unknown_Order_c__Marine_Group_I; f__Unknown_Family_o__Unknown_Order_c__Marine_Group_I; g__Candidatus_Nitrosopelagicus; s__uncultured_archaeon_g__Candidatus_Nitrosopelagicus |
|  | OTU10082 | 55 | 100.72 | c__Alphaproteobacteria; o__Rhodospirillales; f__Rhodospirillaceae; g__norank_f__Rhodospirillaceae; s__uncultured_marine_bacterium_g__norank_f__Rhodospirillaceae |
|  | OTU9455 | 58 | 129.19 | c__Alphaproteobacteria; o__Rickettsiales; f__Mitochondria; g__norank_f__Mitochondria; s__marine_metagenome_g__norank_f__Mitochondria |
|  | OTU2689 | 25 | 133.38 | c__Cyanobacteria; o__SubsectionI; f__FamilyI_o__SubsectionI; g__Synechococcus; s__unclassified_g__Synechococcus |
|  | OTU4860 | 32 | 136.21 | c__Alphaproteobacteria; o__SAR11_clade; f__Deep_1; g__norank_f__Deep_1; s__uncultured_bacterium_g__norank_f__Deep_1 |
|  | OTU3569 | 43 | 141.98 | c__Flavobacteriia; o__Flavobacteriales; f__Flavobacteriaceae; g__NS5_marine_group; s__uncultured_bacterium_g__NS5_marine_group |
|  | OTU3734 | 63 | 162.38 | c__Alphaproteobacteria; o__Rhodospirillales; f__Rhodospirillaceae; g__Magnetospira; s__unclassified_g__Magnetospira |
|  | OTU9276 | 48 | 168.35 | c__Thermoplasmata; o__Thermoplasmatales; f__Marine_Group_II; g__norank_f__Marine_Group_II; s__uncultured_marine_group_II_euryarchaeote_HF70_59C08 |
| Summer-PA | OTU10903 | 32 | 16.54 | c__Marine_Group_I; o__Unknown_Order_c__Marine_Group_I; f__Unknown_Family_o__Unknown_Order_c__Marine_Group_I; g__Candidatus_Nitrosopelagicus; s__uncultured_archaeon_g__Candidatus_Nitrosopelagicus |
|  | OTU12060 | 38 | 37.75 | c__Marine_Group_I; o__unclassified_c__Marine_Group_I; f__unclassified_c__Marine_Group_I; g__unclassified_c__Marine_Group_I; s__unclassified_c__Marine_Group_I |
|  | OTU10082 | 29 | 40.84 | c__Alphaproteobacteria; o__Rhodospirillales; f__Rhodospirillaceae; g__norank_f__Rhodospirillaceae; s__uncultured_marine_bacterium_g__norank_f__Rhodospirillaceae |
|  | OTU3720 | 25 | 62.27 | c__Flavobacteriia; o__Flavobacteriales; f__NS9_marine_group; g__norank_f__NS9_marine_group; s__uncultured_Flavobacteriales_bacterium_g__norank_f__NS9_marine_group |
|  | OTU7425 | 42 | 65.93 | c__Marine_Group_I; o__Unknown_Order_c__Marine_Group_I; f__Unknown_Family_o__Unknown_Order_c__Marine_Group_I; g__Candidatus_Nitrosopelagicus; s__unclassified_g__Candidatus_Nitrosopelagicus |
|  | OTU1507 | 30 | 90.03 | c__Flavobacteriia; o__Flavobacteriales; f__Cryomorphaceae; g__unclassified_f__Cryomorphaceae; s__unclassified_f__Cryomorphaceae |
|  | OTU10487 | 30 | 102.87 | c__Gammaproteobacteria; o__Xanthomonadales; f__JTB255_marine_benthic_group; g__norank_f__JTB255_marine_benthic_group; s__uncultured_bacterium_g__norank_f__JTB255_marine_benthic_group |
|  | OTU7458 | 32 | 108.13 | c__Gammaproteobacteria; o__Gammaproteobacteria_Incertae_Sedis; f__Unknown_Family_o__Gammaproteobacteria_Incertae_Sedis; g__norank_f__Unknown_Family_o__Gammaproteobacteria_Incertae_Sedis; s__uncultured_sediment_bacterium_g__norank_f__Unknown_Family |
|  | OTU1762 | 50 | 124.82 | c__Marine_Group_I; o__Unknown_Order_c__Marine_Group_I; f__Unknown_Family_o__Unknown_Order_c__Marine_Group_I; g__Candidatus_Nitrosopelagicus; s__marine_metagenome_g__Candidatus_Nitrosopelagicus |
|  | OTU11941 | 44 | 125.51 | c__Flavobacteriia; o__Flavobacteriales; f__Flavobacteriaceae; g__NS2b_marine_group; s__uncultured_bacterium_g__NS2b_marine_group |
|  | OTU6233 | 29 | 136.13 | c__Gammaproteobacteria; o__Cellvibrionales; f__Halieaceae; g__Haliea; s__uncultured_bacterium_g__Haliea |
|  | OTU11027 | 50 | 155.66 | c__Thermoplasmata; o__Thermoplasmatales; f__Marine_Group_II; g__norank_f__Marine_Group_II; s__uncultured_marine_group_II_euryarchaeote |
|  | OTU11328 | 26 | 172.72 | c__Gammaproteobacteria; o__Xanthomonadales; f__JTB255_marine_benthic_group; g__norank_f__JTB255_marine_benthic_group; s__unclassified_g__norank_f__JTB255_marine_benthic_group |
|  | OTU9886 | 30 | 202.11 | c__Alphaproteobacteria; o__Rhodospirillales; f__Rhodospirillaceae; g__norank_f__Rhodospirillaceae; s__unclassified_g__norank_f__Rhodospirillaceae |
|  | OTU9570 | 38 | 207.06 | c__Actinobacteria; o__Acidimicrobiales; f__Sva0996_marine_group; g__norank_f__Sva0996_marine_group; s__uncultured_actinobacterium_HF0130_15N16 |
|  | OTU9867 | 26 | 207.31 | c__Gammaproteobacteria; o__BD7-8_marine_group; f__norank_o__BD7-8_marine_group; g__norank_o__BD7-8_marine_group; s__uncultured_gamma_proteobacterium_g__norank_o__BD7-8_marine_group |
|  | OTU1995 | 36 | 243.08 | c__Gammaproteobacteria; o__Xanthomonadales; f__JTB255_marine_benthic_group; g__norank_f__JTB255_marine_benthic_group; s__unclassified_g__norank_f__JTB255_marine_benthic_group |
|  | OTU9852 | 41 | 262.32 | c__Gammaproteobacteria; o__norank_c__Gammaproteobacteria; f__norank_c__Gammaproteobacteria; g__norank_c__Gammaproteobacteria; s__unclassified_g__norank_c__Gammaproteobacteria |
| Winter-FL | OTU3647 | 39 | 42.15 | c__Alphaproteobacteria; o__Rhizobiales; f__OCS116_clade; g__norank_f__OCS116_clade; s__unclassified_g__norank_f__OCS116_clade |
|  | OTU3109 | 37 | 55.15 | c__Alphaproteobacteria; o__Rhodospirillales; f__Rhodospirillaceae; g__norank_f__Rhodospirillaceae; s__unclassified_g__norank_f__Rhodospirillaceae |
|  | OTU12508 | 35 | 57.27 | c__Alphaproteobacteria; o__SAR11_clade; f__Surface_1; g__unclassified_f__Surface_1; s__unclassified_f__Surface_1 |
|  | OTU4368 | 36 | 91.45 | c__Alphaproteobacteria; o__Rickettsiales; f__SAR116_clade; g__norank_f__SAR116_clade; s__uncultured_Oceanibaculum_sp._g__norank_f__SAR116_clade |
|  | OTU4310 | 41 | 112.93 | c__Alphaproteobacteria; o__Rhodospirillales; f__Rhodospirillaceae; g__norank_f__Rhodospirillaceae; s__unidentified_marine_bacterioplankton_g__norank_f__Rhodospirillaceae |
|  | OTU11963 | 38 | 128.83 | c__Alphaproteobacteria; o__SAR11_clade; f__Surface_2; g__norank_f__Surface_2; s__uncultured_bacterium_g__norank_f__Surface_2 |
|  | OTU9303 | 37 | 139.86 | c__Alphaproteobacteria; o__Rhodospirillales; f__Rhodospirillaceae; g__norank_f__Rhodospirillaceae; s__unclassified_g__norank_f__Rhodospirillaceae |
|  | OTU1148 | 37 | 158.76 | c__Alphaproteobacteria; o__SAR11_clade; f__Surface_1; g__unclassified_f__Surface_1; s__unclassified_f__Surface_1 |
|  | OTU11025 | 55 | 170.62 | c__Gammaproteobacteria; o__Oceanospirillales; f__Oceanospirillaceae; g__Pseudohongiella; s__marine_metagenome_g__Pseudohongiella |
|  | OTU5191 | 52 | 178.86 | c__Gammaproteobacteria; o__Oceanospirillales; f__OM182_clade; g__norank_f__OM182_clade; s__uncultured_gamma_proteobacterium_g__norank_f__OM182_clade |
|  | OTU3638 | 40 | 186.81 | c__Alphaproteobacteria; o__Rickettsiales; f__SAR116_clade; g__norank_f__SAR116_clade; s__unclassified_g__norank_f__SAR116_clade |
|  | OTU3050 | 50 | 186.91 | c__Alphaproteobacteria; o__Rhodospirillales; f__unclassified_o__Rhodospirillales; g__unclassified_o__Rhodospirillales; s__unclassified_o__Rhodospirillales |
|  | OTU5677 | 48 | 191.85 | c__Alphaproteobacteria; o__Rickettsiales; f__S25-593; g__norank_f__S25-593; s__unclassified_g__norank_f__S25-593 |
| Winter-PA | OTU9989 | 159 | 44.94 | c__Gammaproteobacteria; o__Xanthomonadales; f__JTB255_marine_benthic_group; g__norank_f__JTB255_marine_benthic_group; s__unclassified_g__norank_f__JTB255_marine_benthic_group |
|  | OTU3596 | 157 | 52.37 | c__Betaproteobacteria; o__Methylophilales; f__Methylophilaceae; g__OM43_clade; s__uncultured_bacterium_g__OM43_clade |
|  | OTU1498 | 162 | 52.68 | c__Actinobacteria; o__Acidimicrobiales; f__Sva0996_marine_group; g__norank_f__Sva0996_marine_group; s__uncultured_bacterium_g__norank_f__Sva0996_marine_group |
|  | OTU6211 | 157 | 54.02 | c__Gammaproteobacteria; o__Xanthomonadales; f__JTB255_marine_benthic_group; g__norank_f__JTB255_marine_benthic_group; s__unclassified_g__norank_f__JTB255_marine_benthic_group |
|  | OTU3571 | 160 | 56.34 | c__Alphaproteobacteria; o__Rhodospirillales; f__Rhodospirillaceae; g__AEGEAN-169_marine_group; s__unclassified_g__AEGEAN-169_marine_group |
|  | OTU7306 | 160 | 57.61 | c__Alphaproteobacteria; o__Rhizobiales; f__Hyphomicrobiaceae; g__Filomicrobium; s__uncultured_alpha_proteobacterium_g__Filomicrobium |
|  | OTU3645 | 161 | 59.19 | c__Alphaproteobacteria; o__Rhodospirillales; f__Rhodospirillaceae; g__OM75_clade; s__uncultured_bacterium_g__OM75_clade |
|  | OTU3619 | 162 | 66.80 | c__Alphaproteobacteria; o__Rhodobacterales; f__Rhodobacteraceae; g__unclassified_f__Rhodobacteraceae; s__unclassified_f__Rhodobacteraceae |
|  | OTU4675 | 160 | 68.01 | c__Alphaproteobacteria; o__Rickettsiales; f__S25-593; g__norank_f__S25-593; s__unclassified_g__norank_f__S25-593 |

*^a^* FL: Free-living; PA: Particle-associated.

**Table S9** Correlations and topological properties of co-occurrence networks of microbial communities from different seasons and lifestyles.

| Network parameters | SummerFL | | SummerPA | WinterFL | WinterPA |
| --- | --- | --- | --- | --- | --- |
| Nodes | | 369 | 484 | 301 | 278 |
| Edges | | 2940 | 3245 | 2751 | 12040 |
| Proportion of positive correlation | | 83.5% | 98.3% | 99% | 96.4% |
| Average degree | | 15.93496 | 13.381 | 18.27907 | 86.619 |
| Average network distance | | 4.264 | 5.277 | 3.719 | 2.401 |
| Average clustering Coefficient | | 0.508 | 0.468 | 0.577 | 0.792 |
| Average weighted degree | | 8.419 | 9.968 | 13.938 | 63.828 |
| Diameter | | 13 | 22 | 12 | 10 |
| Modularity index | | 0.62 | 0.598 | 0.478 | 0.099 |
| The proportion of different taxa | | Alphaproteobacteria (34.96%)  Gammaproteobacteria (21.95%)  Flavobacteriia (21.14%)  Actinobacteria (7.03%)  Thermoplasmata (4.88%)  Marine_Group_I (4.07%)  Cyanobacteria (4.07%)  *Bacilli* (1.9%) | Alphaproteobacteria (28.87%)  Gammaproteobacteria (26.8%)  Flavobacteriia (18.97%)  Cyanobacteria (10.1%)  Actinobacteria (7.63%)  Thermoplasmata (2.89%)  Betaproteobacteria (2.68%)  Marine_Group_I (2.06%) | Alphaproteobacteria (38.87%)  Gammaproteobacteria (22.26%)  Actinobacteria (9.3%)  Flavobacteriia (8.31%)  Cyanobacteria (7.97%)  Thermoplasmata (5.65%)  *Bacilli* (4.98%)  Marine_Group_I (2.66%) | Alphaproteobacteria (32.37%)  Gammaproteobacteria (29.5%)  Cyanobacteria (14.39%)  Actinobacteria (9.71%)  Flavobacteriia (6.47%)  Betaproteobacteria (2.88%)  Thermoplasmata (2.52%)  Marine_Group_I (2.16%) |

*^a^* FL: Free-living; PA: Particle-associated.

**Table S10** Correlation and topological properties of microbial community co-occurrence networks in nearshore and offshore sites of the PN section.

| Network Parameters | SummerFL-P1P2 | SummerFL-P5P6 | SummePA-P1P2 | SummerPA-P5P6 | WinterFL-P1P2 | WinterFL-P5P6P7 | WinterPA-P1P2 | WinterPA-P5P6P7 |
| --- | --- | --- | --- | --- | --- | --- | --- | --- |
| Nodes | 338 | 377 | 488 | 531 | 351 | 341 | 478 | 170 |
| Edges | 7638 | 8890 | 9889 | 6887 | 3612 | 5782 | 10467 | 1277 |
| Proportion of positive correlation | 95.8% | 69.3% | 99.5% | 99.8% | 99.6% | 96.3% | 99.3% | 97.6% |
| Average degree | 40.243 | 47.126 | 40.529 | 25.94 | 20.581 | 33.912 | 43.795 | 15.042 |
| Average network distance | 2.65 | 2.565 | 2.999 | 3.273 | 3.356 | 3.032 | 2.948 | 3.367 |
| Average clustering Coefficient | 0.626 | 0.566 | 0.569 | 0.486 | 0.508 | 0.589 | 0.565 | 0.643 |
| Average weighted degree | 33.807 | 28.291 | 33.768 | 20.382 | 34.373 | 25.188 | 36.704 | 11.31 |
| Diameter | 7 | 7 | 6 | 10 | 7 | 9 | 9 | 9 |
| Modularity index | 0.567 | 1.38 | 0.479 | 0.51 | 0.587 | 0.354 | 0.415 | 0.377 |
| The proportion of different taxa | Alphaproteobacteria (35.5%)  Flavobacteriia (20.41%)  Gammaproteobacteria (20.12%)  Actinobacteria (10.65%)  Thermoplasmata (5.03%)  Cyanobacteria (4.73%)  Marine_Group_I (3.55%) | Alphaproteobacteria (29.71%)  Gammaproteobacteria (27.46%)  Flavobacteria (19.26%)  Actinobacteria (8.81%)  Cyanobacteria (6.56%)  Bacilli (3.89%)  Thermoplasmata (2.25%)  Marine_Group_I (2.05%) | Alphaproteobacteria (38.2%)  Gammaproteobacteria (20.95%)  Flavobacteria (18.04%)  Actinobacteria (7.96%)  Thermoplasmata (4.51%)  Marine_Group_I (3.98%)  Cyanobacteria (3.98%)  *Bacilli* (2.39%) | Alphaproteobacteria (30.13%)  Gammaproteobacteria (24.86%)  Flavobacteria (17.14%)  Cyanobacteria (10.55%)  Actinobacteria (6.56%)  Bacilli (4.33%)  Thermoplasmata (1.88%)  Marine_Group_I (1.51%) | Alphaproteobacteria (36.75%)  Gammaproteobacteria (23.08%)  Actinobacteria (11.97%)  Cyanobacteriia (8.83%)  Flavobacteria (7.41%)  Betaproteobacteria (5.41%)  Thermoplasmata (4.27%)  Marine_Group_I (2.28%) | Alphaproteobacteria (31.17%)  Gammaproteobacteria (29.08%)  Actinobacteria (14.85%)  Cyanobacteriia (8.83%)  Flavobacteria (7.32%)  Betaproteobacteria (5.23%)  Thermoplasmata (2.93%)  Marine_Group_I (1.88%) | Alphaproteobacteria (39%)  Gammaproteobacteria (21.99%)  Actinobacteria (12.02%)  Flavobacteria (9.09%)  Cyanobacteriia (6.16%)  Thermoplasmata (5.28%)  Marine_Group_I (3.52%)  Betaproteobacteria (2.93%) | Cyanobacteriia (25.29%)  Gammaproteobacteria (21.99%)  Alphaproteobacteria (21.76%)  Marine_Group_I (7.65%)  Actinobacteria (6.47%)  Flavobacteria (5.88%)  Thermoplasmata (4.12%)  Betaproteobacteria (4.12%) |

*^a^* FL: Free-living; PA: Particle-associated.

**TableS11** Correlation between *Ralstonia* and other OTUs in the co-occurrence network.

| Group | source | | target | class | genus | correlation |
| --- | --- | --- | --- | --- | --- | --- |
| winterP1P2FL | OTU12867 | g__Ralstonia | OTU4242 | c__Actinobacteria | g__Euzebya | positive |
|  | OTU12867 | g__Ralstonia | OTU1146 | c__Alphaproteobacteria | g__Loktanella | positive |
|  | OTU12867 | g__Ralstonia | OTU4276 | c__Alphaproteobacteria | g__Paracoccus | positive |
|  | OTU12867 | g__Ralstonia | OTU6374 | c__Alphaproteobacteria | g__Rhizobium | positive |
|  | OTU8237 | g__Ralstonia | OTU6363 | c__Flavobacteriia | g__Salinirepens | positive |
|  | OTU12867 | g__Ralstonia | OTU13201 | c__Gammaproteobacteria | g__Acidibacter | positive |
|  | OTU8237 | g__Ralstonia | OTU4975 | c__Gammaproteobacteria | g__Coxiella | positive |
| winterP1P2PA | OTU12867 | g__Ralstonia | OTU10283 | c__Alphaproteobacteria | g__unclassified_f__Rhodobacteraceae | positive |
|  | OTU12867 | g__Ralstonia | OTU3834 | c__Alphaproteobacteria | g__Loktanella | positive |
|  | OTU12867 | g__Ralstonia | OTU8074 | c__Alphaproteobacteria | g__norank_o__Alphaproteobacteria_Incertae_Sedis | positive |
|  | OTU12867 | g__Ralstonia | OTU13028 | c__Betaproteobacteria | g__Limnobacter | positive |
|  | OTU12867 | g__Ralstonia | OTU7349 | c__Gammaproteobacteria | g__norank_o__KI89A_clade | positive |
|  | OTU12867 | g__Ralstonia | OTU12507 | c__Gammaproteobacteria | g__norank_f__SAR86_clade | negative |
|  | OTU12867 | g__Ralstonia | OTU2779 | c__Gammaproteobacteria | g__Psychromonas | negative |
|  | OTU12867 | g__Ralstonia | OTU800 | c__Gammaproteobacteria | g__norank_o__HTA4 | positive |
| winterP5P6P7FL | OTU12867 | g__Ralstonia | OTU8025 | c__Actinobacteria | g__Candidatus_Actinomarina | negative |
|  | OTU12867 | g__Ralstonia | OTU12515 | c__Alphaproteobacteria | g__norank_f__Surface_1 | negative |
|  | OTU12867 | g__Ralstonia | OTU4106 | c__Alphaproteobacteria | g__norank_f__Rhodobacteraceae | negative |
|  | OTU12867 | g__Ralstonia | OTU9033 | c__Alphaproteobacteria | g__unclassified_f__Rhodobacteraceae | negative |
|  | OTU12867 | g__Ralstonia | OTU9359 | c__Alphaproteobacteria | g__AEGEAN-169_marine_group | negative |
|  | OTU12867 | g__Ralstonia | OTU1148 | c__Alphaproteobacteria | g__unclassified_f__Surface_1 | negative |
|  | OTU12867 | g__Ralstonia | OTU2369 | c__Alphaproteobacteria | g__norank_f__PS1_clade | negative |
|  | OTU12867 | g__Ralstonia | OTU9635 | c__Alphaproteobacteria | g__norank_f__S25-593 | negative |
|  | OTU12867 | g__Ralstonia | OTU3602 | c__Alphaproteobacteria | g__Candidatus_Puniceispirillum | negative |
|  | OTU12867 | g__Ralstonia | OTU5314 | c__Alphaproteobacteria | g__Ascidiaceihabitans | negative |
|  | OTU12867 | g__Ralstonia | OTU3647 | c__Alphaproteobacteria | g__norank_f__OCS116_clade | negative |
|  | OTU12867 | g__Ralstonia | OTU3100 | c__Alphaproteobacteria | g__norank_f__AT-s3-44 | negative |
|  | OTU12867 | g__Ralstonia | OTU9303 | c__Alphaproteobacteria | g__norank_f__Rhodospirillaceae | negative |
|  | OTU12867 | g__Ralstonia | OTU10460 | c__Alphaproteobacteria | g__OM75_clade | negative |
|  | OTU12867 | g__Ralstonia | OTU7552 | c__Alphaproteobacteria | g__unclassified_f__Rhodobacteraceae | negative |
|  | OTU12867 | g__Ralstonia | OTU6374 | c__Alphaproteobacteria | g__Rhizobium | positive |
|  | OTU12867 | g__Ralstonia | OTU8814 | c__Betaproteobacteria | g__Burkholderia-Paraburkholderia | positive |
|  | OTU12867 | g__Ralstonia | OTU3596 | c__Betaproteobacteria | g__OM43_clade | negative |
|  | OTU12867 | g__Ralstonia | OTU9221 | c__Betaproteobacteria | g__norank_f__Hydrogenophilaceae | negative |
|  | OTU12867 | g__Ralstonia | OTU2691 | c__Cyanobacteria | g__unclassified_f__FamilyI_o__SubsectionI | negative |
|  | OTU12867 | g__Ralstonia | OTU2689 | c__Cyanobacteria | g__Synechococcus | negative |
|  | OTU12867 | g__Ralstonia | OTU5590 | c__Cyanobacteria | g__Synechococcus | negative |
|  | OTU12867 | g__Ralstonia | OTU8894 | c__Flavobacteriia | g__NS5_marine_group | negative |
|  | OTU12867 | g__Ralstonia | OTU4904 | c__Flavobacteriia | g__NS4_marine_group | negative |
|  | OTU12867 | g__Ralstonia | OTU1200 | c__Flavobacteriia | g__NS5_marine_group | negative |
|  | OTU12867 | g__Ralstonia | OTU2189 | c__Flavobacteriia | g__NS5_marine_group | negative |
|  | OTU12867 | g__Ralstonia | OTU9745 | c__Flavobacteriia | g__NS4_marine_group | negative |
|  | OTU12867 | g__Ralstonia | OTU3587 | c__Gammaproteobacteria | g__norank_o__E01-9C-26_marine_group | negative |
|  | OTU12867 | g__Ralstonia | OTU1763 | c__Gammaproteobacteria | g__ZD0417_marine_group | negative |
|  | OTU12867 | g__Ralstonia | OTU9025 | c__Gammaproteobacteria | g__norank_f__SAR86_clade | negative |
|  | OTU12867 | g__Ralstonia | OTU12512 | c__Gammaproteobacteria | g__norank_f__SAR86_clade | negative |
|  | OTU12867 | g__Ralstonia | OTU3641 | c__Gammaproteobacteria | g__norank_f__SAR86_clade | negative |
|  | OTU12867 | g__Ralstonia | OTU2509 | c__Gammaproteobacteria | g__norank_f__SAR86_clade | negative |
|  | OTU12867 | g__Ralstonia | OTU3630 | c__Gammaproteobacteria | g__Thiothrix | negative |
|  | OTU12867 | g__Ralstonia | OTU2508 | c__Gammaproteobacteria | g__OM60_NOR5__clade | negative |
|  | OTU12867 | g__Ralstonia | OTU3672 | c__Gammaproteobacteria | g__norank_f__JTB255_marine_benthic_group | negative |
|  | OTU12867 | g__Ralstonia | OTU3048 | c__Gammaproteobacteria | g__Thiothrix | negative |
|  | OTU12867 | g__Ralstonia | OTU3607 | c__Gammaproteobacteria | g__norank_f__OM182_clade | negative |
|  | OTU12867 | g__Ralstonia | OTU2909 | c__Gammaproteobacteria | g__norank_f__SAR86_clade | negative |
|  | OTU12867 | g__Ralstonia | OTU13201 | c__Gammaproteobacteria | g__Acidibacter | positive |
|  | OTU12867 | g__Ralstonia | OTU11016 | c__Marine_Group_I | g__Candidatus_Nitrosopelagicus | negative |
|  | OTU12867 | g__Ralstonia | OTU1762 | c__Marine_Group_I | g__Candidatus_Nitrosopelagicus | negative |
|  | OTU12867 | g__Ralstonia | OTU11691 | c__Marine_Group_I | g__Candidatus_Nitrosopelagicus | negative |
|  | OTU12867 | g__Ralstonia | OTU3177 | c__Marine_Group_I | g__Candidatus_Nitrosopumilus | negative |
|  | OTU12867 | g__Ralstonia | OTU5108 | c__Marine_Group_I | g__Candidatus_Nitrosopumilus | negative |
|  | OTU12867 | g__Ralstonia | OTU10905 | c__Thermoplasmata | g__norank_f__Marine_Group_II | negative |
|  | OTU12867 | g__Ralstonia | OTU5676 | c__Thermoplasmata | g__norank_f__Marine_Group_II | negative |
| winterP5P6P7PA | OTU12867 | g__Ralstonia | OTU8155 | c__Actinobacteria | g__Gordonia | negative |
|  | OTU12867 | g__Ralstonia | OTU9597 | c__Actinobacteria | g__Candidatus_Actinomarina | negative |
|  | OTU12867 | g__Ralstonia | OTU10889 | c__Alphaproteobacteria | g__norank_f__Surface_1 | negative |
|  | OTU12867 | g__Ralstonia | OTU12515 | c__Alphaproteobacteria | g__norank_f__Surface_1 | negative |
|  | OTU12867 | g__Ralstonia | OTU11015 | c__Alphaproteobacteria | g__norank_f__Deep_1 | negative |
|  | OTU12867 | g__Ralstonia | OTU8159 | c__Alphaproteobacteria | g__norank_f__Surface_2 | negative |
|  | OTU12867 | g__Ralstonia | OTU11022 | c__Alphaproteobacteria | g__norank_f__Surface_2 | negative |
|  | OTU12867 | g__Ralstonia | OTU4891 | c__Alphaproteobacteria | g__norank_f__Surface_2 | negative |
|  | OTU12867 | g__Ralstonia | OTU11174 | c__Betaproteobacteria | g__Alcaligenes | negative |
|  | OTU12867 | g__Ralstonia | OTU12509 | c__Cyanobacteria | g__Synechococcus | negative |
|  | OTU12867 | g__Ralstonia | OTU4302 | c__Cyanobacteria | g__Synechococcus | negative |
|  | OTU12867 | g__Ralstonia | OTU9602 | c__Gammaproteobacteria | g__unclassified_f__Oceanospirillaceae | negative |
|  | OTU12867 | g__Ralstonia | OTU3624 | c__Gammaproteobacteria | g__Halomonas | negative |
|  | OTU12867 | g__Ralstonia | OTU9131 | c__Thermoplasmata | g__norank_f__Marine_Group_II | negative |
